# LncRNA PTENP1/miR-21/PTEN Axis Modulates EMT and Drug Resistance in Cancer: Dynamic Boolean Modeling for Cell Fates in DNA Damage Response

**DOI:** 10.3390/ijms25158264

**Published:** 2024-07-29

**Authors:** Shantanu Gupta, Daner A. Silveira, Pedro R. Lorenzoni, Jose Carlos M. Mombach, Ronaldo F. Hashimoto

**Affiliations:** 1 Instituto de Matemática e Estatística, Departamento de Ciência da Computação, Universidade de São Paulo, Rua do Matão 1010, São Paulo 05508-090, SP, Brazil; ronaldo@ime.usp.br; 2 Children’s Cancer Institute, Porto Alegre 90620-110, RS, Brazil; d.silveira.bioinfo@ici.ong; 3 Departamento de Física, Universidade Federal de Santa Maria, Santa Maria 97105-900, RS, Brazil; pedro.lorenzoni@acad.ufsm.br (P.R.L.); jcmombach@ufsm.br (J.C.M.M.)

**Keywords:** miR-21, PTEN, lncRNA PTENP1, epithelial-to-mesenchymal transition, drug resistance, senescence, cell cycle arrest, apoptosis, autophagy, feedback loops

## Abstract

It is well established that microRNA-21 (miR-21) targets phosphatase and tensin homolog (PTEN), facilitating epithelial-to-mesenchymal transition (EMT) and drug resistance in cancer. Recent evidence indicates that PTEN activates its pseudogene-derived long non-coding RNA, PTENP1, which in turn inhibits miR-21. However, the dynamics of PTEN, miR-21, and PTENP1 in the DNA damage response (DDR) remain unclear. Thus, we propose a dynamic Boolean network model by integrating the published literature from various cancers. Our model shows good agreement with the experimental findings from breast cancer, hepatocellular carcinoma (HCC), and oral squamous cell carcinoma (OSCC), elucidating how DDR activation transitions from the intra-S phase to the G2 checkpoint, leading to a cascade of cellular responses such as cell cycle arrest, senescence, autophagy, apoptosis, drug resistance, and EMT. Model validation underscores the roles of PTENP1, miR-21, and PTEN in modulating EMT and drug resistance. Furthermore, our analysis reveals nine novel feedback loops, eight positive and one negative, mediated by PTEN and implicated in DDR cell fate determination, including pathways related to drug resistance and EMT. Our work presents a comprehensive framework for investigating cellular responses following DDR, underscoring the therapeutic potential of targeting PTEN, miR-21, and PTENP1 in cancer treatment.

## 1. Introduction

Long non-coding RNAs (lncRNAs) and microRNAs (miRNAs) are pivotal in numerous biological processes, including cell cycle progression, apoptosis, and tumorigenesis [1]. In cancer, miRNAs serve dual roles as tumor suppressors or oncogenes, exerting significant influence [1]. MicroRNA-21 (miR-21) plays a crucial role in various cancers, including breast cancer, non-small cell lung cancer (NSCLC), prostate cancer, hepatocellular carcinoma (HCC), and oral squamous cell carcinoma (OSCC) by targeting PTEN, thereby affecting EMT and drug resistance through AKT signaling pathways [2,3]. Database analyses and PCR data from these cancers further underscore the significant role of miR-21 in modulating cell fates and contributing to oncogenic processes [4,5]. The lncRNA PTENP1 (hereafter referred to as PTENP1) acts as a competing endogenous RNA, sequestering miR-21 and protecting PTEN from degradation [6]. Research highlights the importance of PTENP1 across various cancers, including prostate cancer [7], HCC [8], OSCC [9], and clear cell renal cell carcinoma (ccRCC) [6] in regulating the miR-21/PTEN axis, thereby influencing EMT, drug resistance, and other cellular functions. Studies also indicate that DNA-damaging agents upregulate PTEN by downregulating miR-21, leading to G1/S arrest and apoptosis [10,11].

The intricate relationship between drug resistance and epithelial-to-mesenchymal transition (EMT) complicates cancer treatment strategies, where EMT enhances resistance to anti-cancer therapies [12]. Senescence, a protective mechanism against tumorigenesis, involves growth arrest and permanent cell cycle exit, regulated in part by the cyclin-dependent kinase inhibitor 1A (p21) and influenced by the mammalian target of the rapamycin (mTOR) pathway [13,14]. The cell cycle is tightly regulated by cyclin-dependent kinases (CDKs) and their partners to ensure precise phase transitions. Key regulators such as CDK4/6-cyclin D, CDK2-cyclin E, CDK2-cyclin A2, and CDK1-cyclin B orchestrate these transitions, while inhibitory factors, like p21, modulate cell cycle progression by targeting CDK-cyclin complexes [15]. Protein kinase B (AKT) phosphorylates p21, promoting cell cycle progression and inhibiting apoptosis [16]. PTEN, a critical tumor suppressor, regulates AKT activity, thereby influencing p21 expression and impacting cell cycle dynamics, DNA damage response, autophagy, and apoptosis [17].

Additional studies highlight PTEN’s role in inducing G2/M arrest in breast cancer cells, where it inhibits Cdc25C, thereby activating the ATM/p53 pathway [18]. PTENP1 also contributes to intra-S to G2/M phase arrest by targeting CDK2-cyclin A2 in breast cancer cells, suggesting its relevance in cell cycle regulation [19]. However, the precise molecular mechanism involving PTENP1/miR-21 and the PTEN axis remains unclear. Based on the aforementioned insights, we propose a dynamic Boolean network model that combines PTENP1, miR-21, and protein signaling pathways. We aim to uncover how these non-coding RNAs (ncRNAs) impact EMT, drug resistance, and cellular outcomes, such as cell cycle arrest, senescence, autophagy, and apoptosis, in cancer.

## 2. Results

### 2.1. Fixed Point Analysis of the Wild-Type Scenario in the Boolean Network

**Figure 1:**
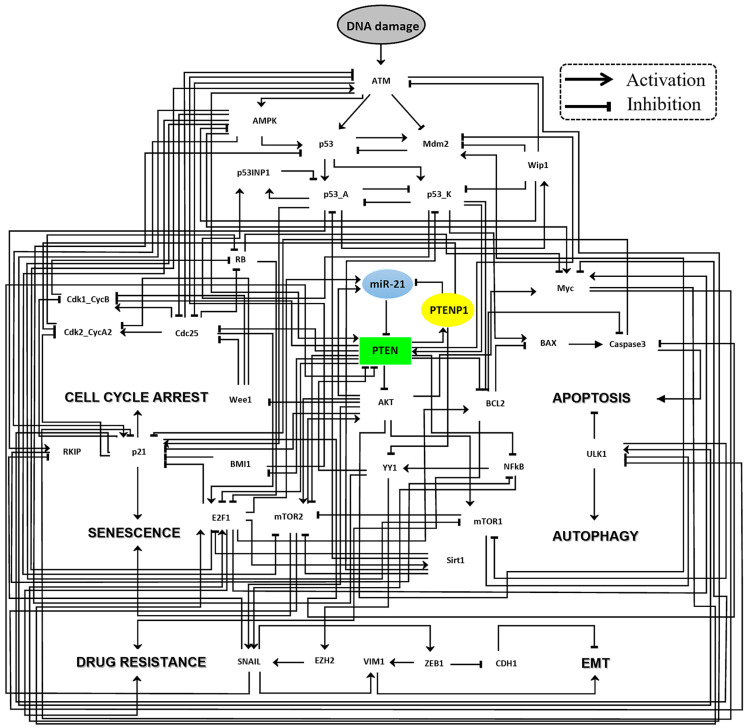
The network that orchestrates EMT, drug resistance, and cell fate dynamics in cancer cells through the PTEN/PTENP1 and miR-21 axis. Solid black lines with arrowheads indicate positive or regulatory interactions, while solid black lines with hammerheads denote negative interactions or regulatory influences. The color coding of the nodes highlights their roles: signaling proteins are in white, except for the PTEN rectangular node (in green). The lncRNA PTENP1 (in yellow) and miR-21 (in blue) are represented in oval nodes. A gray oval node signifies DNA damage as the initiating input. The outcomes of the model such as cycle arrest, senescence, autophagy, apoptosis, drug resistance, and EMT are clearly labeled. For an in-depth explanation of network components, their complete names, and the biological logic behind the connections and their regulators, see Table S1, while the proposed molecular mechanism can be found in File S1.

The network comprises forty-three signaling components, including one miRNA (miR-21) and one lncRNA (PTENP1). DNA damage is also incorporated as a single input with two possible states: “ON” and “OFF.” Additionally, there are six outputs to the model, representing drug resistance, EMT, cell cycle arrest, senescence, apoptosis, and autophagy. Furthermore, the network contains 117 direct connections among these signaling components (refer to Figure 1 for detailed visualization).

**Figure 2:**
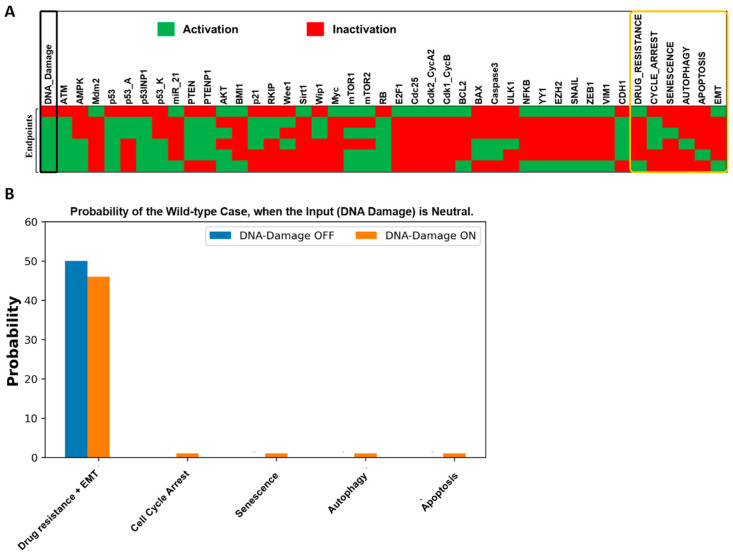
Dynamics of the wild-type case network illustrating six fixed points or endpoints. (**A**) The left-most column depicts DNA damage levels highlighted in the black box, while the right-most column presents the model outputs: drug resistance, cell cycle arrest, senescence, autophagy, apoptosis, and EMT, which are highlighted in the orange box. Each line represents a fixed point corresponding to the input. Red cells denote the inactivation of the corresponding molecule or phenotype, whereas green cells denote the activation of the corresponding molecule or phenotype (value 1). (**B**) A Monte Carlo simulation (100,000 runs) for determining each fixed point or phenotype in a wild-type scenario under a “Neutral” input. For more detail, see Section 2.1.

We elucidate the dynamics of the wild-type case network, revealing six fixed points (also known as endpoints or stable states). For more detail, see Figure 2A, where each line represents a fixed point corresponding to the input. Purple and gray cells denote molecule activation and deactivation, facilitating the identification of trapped molecules at each fixed point. Our network comprises a single input and DNA damage, toggling between “ON” and “OFF” states. Among the six fixed points, one occurs upon input deactivation, while the remaining five manifest in its presence. In Figure 2A, the first fixed point depicts a proliferative state in the absence of an external input. Here, only cell cycle regulators are active, while tumor suppressors and cell cycle inhibitors remain dormant. Subsequent fixed points, as illustrated in Figure 2A, are characterized by the presence of DNA damage, representing cell cycle arrest, senescence, autophagy, apoptosis, and EMT, respectively.

To delve deeper, the following two fixed points represent cell cycle arrest and senescence. This occurs through the activation of p53-A, which orchestrates cell cycle arrest alongside p21, while senescence is mediated by p53-A-induced mTOR1/2 activation alongside p21. Following this, the next two distinct pathways of cellular demise emerge: autophagy and apoptosis. The autophagy phenotype involves ULK1 induction coupled with the activation of p53-K, BAX, and Caspase-3. Conversely, apoptosis is characterized by the activation of p53-K, BAX, and Caspase-3 without ULK1 induction. The final fixed point denotes drug resistance and EMT, attributed to the activation of miR-21, BCL2, NFkB, and EMT markers, such as SNAIL, zinc finger E-box-binding homeobox 1 (ZEB1), and Vimentin (VIM). Additionally, as depicted in Figure 2A, the emergence of the cell cycle arrest phenotype aligns with the initiation of senescence and autophagy fixed points. Recent investigations on MCF-7 and MDA-MB-231 cell lines have unveiled the induction of autophagy by Artesunate (ART) through the augmentation of the ULK1/Beclin1 complex [20]. This coincides with ART’s capacity to arrest the cell cycle at the G2/M phase, accompanied by an increase in p21 expression [20]. Thus, these findings suggest that ART-induced cell cycle arrest operates in a manner reliant on the autophagy cascade [20]. 

In our model, “drug resistance” encompasses two distinct states. The first state, observed in the absence of DNA damage, denotes inherent resistance, where cancer cells proliferate despite the absence of external stressors, such as chemotherapy or treatment. In contrast, the second state, occurring in response to DNA damage, represents acquired resistance to therapeutic agents induced by genotoxic stress from treatments, like chemotherapy or radiation. Understanding these distinctions is vital for developing tailored therapeutic strategies to effectively combat drug resistance in cancer treatment.

Moreover, employing a non-deterministic (stochastic) network dynamics approach, two or more specific fixed points are randomly selected from the same initial state when the network input is “ON”, with probabilities not necessarily equivalent. As illustrated in Figure 2B, under “neutral” network input conditions, we observed a 50% occurrence of EMT and drug resistance (when the input is OFF). However, when the input is “ON”, we found a 46% incidence of drug resistance and EMT, with cell cycle arrest, senescence, apoptosis, and autophagy each occurring at only a 1% frequency. These findings, derived from Monte Carlo simulations involving 100,000 runs, underscore that drug resistance and EMT are the predominant outcomes, irrespective of DNA damage status; for more detail, see Figure 2B.

### 2.2. Dynamic Model Validation: In Silico Perturbation against Known Experimental Findings

In various cancers, such as breast cancer [21], HCC [22], and OSCC [23], miR-21 consistently exhibits overexpression, which coincides with the concurrent downregulation of PTEN within the same cancer cells. This dysregulation of the miR-21/PTEN axis contributes to a spectrum of phenotypes associated with cancer progression and therapy resistance across these malignancies. Although research on PTENP1 remains limited, recent studies have shed light on its role as a bona fide target of miR-21, induced by PTEN [6]. This discovery adds another layer of complexity to the regulatory network involving miR-21, PTEN, and PTENP1 in cancer development and progression.

**Figure 3:**
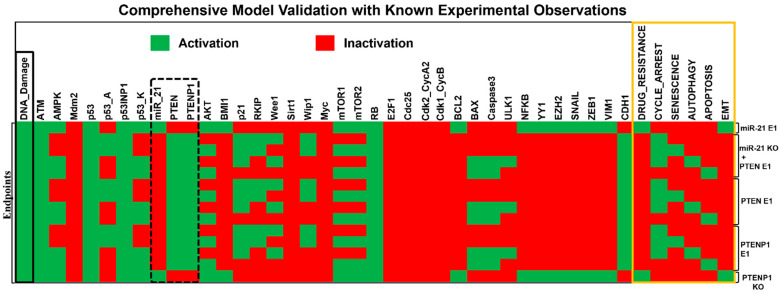
Validation of the Boolean model against known experimental findings: Perturbations were executed on the corresponding molecules, with ectopic expression (E1) representing gain of function (GoF) and knockdown (KO) representing loss of function (LoF). Active network components are illustrated in green cells (ON), indicating activation, while inactive ones are shown in red cells (OFF), indicating inactivation. Fixed points or endpoints were established for specific modeling scenarios, including miR-21 E1, miR-21 KO + PTEN E1, PTEN E1, PTENP1 E1, and PTENP1 KO. These molecules are highlighted with dotted black lines. Input DNA damage is presented in the left column, highlighted in a black box, while model outputs such as drug resistance, cell cycle arrest, senescence, autophagy, apoptosis, and EMT are displayed in the right column, highlighted in an orange box. Each line depicts a single fixed point associated with the input. For more detailed information, refer to Section 2.2.

Our model construction involved integrating a diverse range of studies, capturing various biological phenomena to enhance its accuracy in reflecting real-world scenarios. The studies used for model construction are meticulously detailed in Supplementary Table S1, while the validation process is comprehensively outlined in Table 1. To maintain the integrity of our validation phase, we carefully excluded the studies used for model construction from the validation process, ensuring an independent assessment of our model’s performance against experimental data. This rigorous methodology enhances the reliability and generalizability of our findings. Additionally, we conducted gain of function (GoF) and loss of function (LoF) perturbations, depicted in Figure 3, to further validate the predictive capabilities of our model. The alignment between our model and the experimental findings is briefly summarized in Table 1, with detailed elaboration provided below for clarity.

Initiating with breast cancer cells, miR-21 overexpression inhibits PTEN and promotes cancer progression via the AKT pathway in MCF-7 cells (Wang et al. [21]). Similarly, Ghosh et al. [24] found that miR-21 overexpression in MCF-7, MDA-MB-231, and HeLa cells promotes EMT and drug resistance, suggesting DPA 560 as a potential inhibitor of miR-21 to activate PTEN and suppress these effects (see Figure 3). In breast cancer, PTEN upregulation induced by Erlotinib and Vorinostat inhibits progression through G2/M arrest and apoptosis (Alqosaibi et al. [25]). Chen et al. [19] demonstrated PTENP1’s inhibition of cell proliferation and migration in MCF-7 cells by blocking the S to G2 transition. Gao et al. [26] showed that PTEN and PTENP1 upregulation counters drug resistance and EMT in breast cancer cell lines through PI3K/AKT pathway inhibition and apoptosis induction. However, it is important to note that in this particular study, the authors primarily focused on analyzing miR-20a. Another significant finding comes from De Amicis et al. [27], who revealed that Bergapten-induced PTEN upregulation inhibits AKT signaling and induces autophagy in breast cancer cells (MCF-7, ZR75-1). For further details, refer to Figure 3 and Table 1.

In HCC (HepG2, SK-HEP1, SNU-182, SNU-449, PLC/PRF-5 cell lines), a high expression of miR-21 correlates with proliferation, migration, and invasion due to PTEN knockdown (Meng et al. [22]). In addition, Liu et al. [28] demonstrated that miR-21 inhibition prompts PTEN expression, leading to G2/M arrest and apoptosis in KIM-1, KYN-1, KYN-2, KYN-3, HAK-1A, and HAK-1B HCC cell lines. Furthermore, He et al. [29] found that miR-21 overexpression inhibits autophagy-induced cell death and enhances sorafenib resistance via PTEN/AKT pathway regulation in HepG2 cells.

In OSCC cells (SCC15 and SCC25), Zheng et al. [23] identified elevated miR-21 levels correlating with increased proliferation and invasion, attributed to direct PTEN inhibition. The inhibition of miR-21 significantly suppressed these effects by activating PTEN and inducing apoptosis [23]. Furthermore, Gao et al. [9] further investigated the PTEN/miR-21 and PTENP1 axis in OSCC, revealing PTENP1 as a competing endogenous RNA that sequesters miR-21, promoting PTEN activity. This mechanism suppressed proliferation, and colony formation and induced S-G2/M cell cycle arrest via the AKT pathway [9]. Additionally, similar to findings by Chen et al. [19] in MCF7 breast cancer cells, Gao et al. [9] identified PTENP1’s involvement in CDK2 and cyclin A2 modulation. This action led to the blockade of the cell cycle at the S to G2 phase in SCC-25, Cal-27, HEK 293, and Tca-8113 OSCC cells [9].

**Table 1:** Comprehensive model validation: Model predictions versus established experimental observations of miR-21, PTEN, and PTENP1 in breast, hepatocellular carcinoma (HCC) and oral squamous cell carcinoma (OSCC).

Cancer Type and Their Corresponding Cell Lines	Experimental Observation	References
Breast Cancer (cell lines MCF-7, MDA-MB-231, and ZR75-1). HCC (cell lines HepG2, SK-HEP1, SNU-182, SNU-449, PLC/PRF-5, KIM-1, KYN-1, KYN-2, KYN-3, HAK-1A, and HAK-1B). OSCC (cell lines SCC15, SCC25, Cal-27, HEK 293, and Tca-8113).	Overexpression (E1) of miR-21 inhibits PTEN and activates the AKT pathway, leading to proliferation, drug resistance, and EMT. Knockdown (KO) of miR-21 and upregulation (E1) of PTEN inhibits proliferation, drug resistance, and EMT. Upregulation (E1) of PTEN induces G2/M cell cycle arrest, apoptosis, and autophagy. Knockdown (KO) of PTENP1 induces drug resistance and EMT, while overexpression (E1) inhibits EMT and drug resistance. Overexpression (E1) of PTENP1 induces cell cycle arrest from the S to G2 phase and apoptosis. Overexpression (E1) of PTENP1 is involved in activating autophagy in hepatocellular carcinoma.	[9,19,21,22,23,24,25,26,27,30]

As we can see in Figure 3 and Table 1, based on our comparison, it is evident that the model we developed is capable of producing outcomes similar to those observed in cases of breast, hepatocellular, and oral squamous cell carcinomas. This highlights the effectiveness of our model in uncovering clinically relevant mechanisms that contribute to the development of cancer.

### 2.3. Exploring the Influence of the miR-21/PTEN and PTENP1 Axis on Cell Fate, Drug Resistance, and EMT

**Figure 4:**
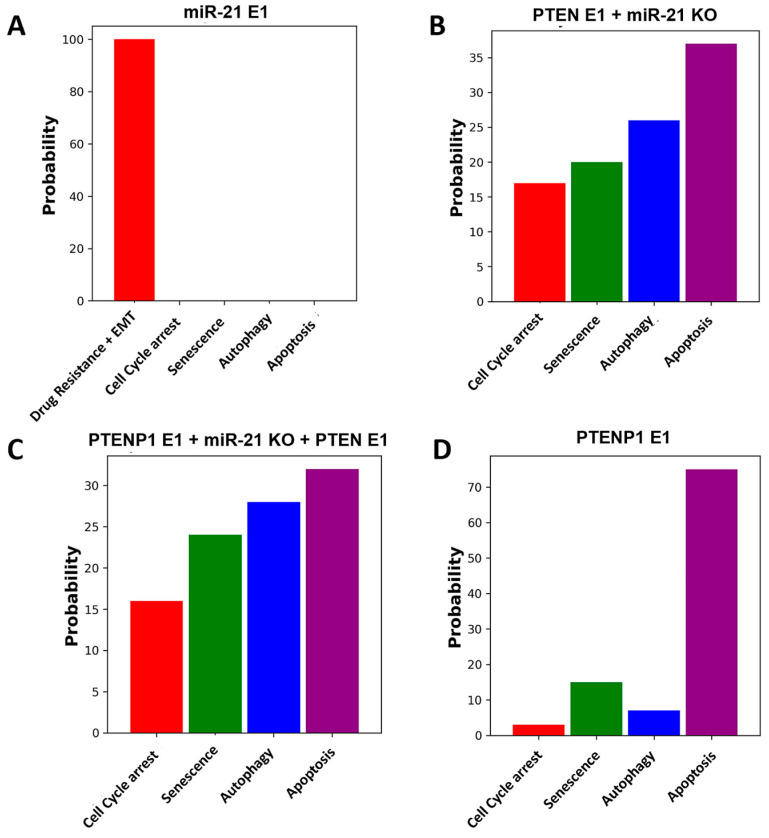
The impact of the PTENP1/miR-21 and PTEN axis on EMT, drug resistance, and cell fate decisions in DDR. Overexpression (E1) represents gain of function (GoF) and knockdown (KO) represents loss of function (LoF) perturbations. (**A**) miR-21 overexpression. (**B**) PTEN overexpression with miR-21 knockdown. (**C**) PTENP1 and PTEN overexpression with miR-21 knockdown. (**D**) PTENP1 overexpression. Each bar represents a cell fate decision, including cell cycle arrest, senescence, autophagy, apoptosis, and EMT + drug resistance. We conducted 100,000 Monte Carlo simulations for each perturbation. For further details, refer to Section 2.3.

We investigated the impact of PTEN/miR-21 and PTENP1 on drug resistance and EMT, thereby influencing cell fate decisions in DDR. Our study aimed to assess the efficacy of four distinct perturbations: overexpressing miR-21, knocking down miR-21 while overexpressing PTEN, knocking down miR-21 while overexpressing both PTEN and PTENP1, and overexpressing PTENP1 alone (see Figure 4). The objective was to ascertain their effects on drug resistance, EMT, and cell fate decisions. To achieve this, we utilized Monte Carlo simulations involving 100,000 runs for each perturbation to determine the influence of these molecules on each phenotype in DDR. 

Our findings, as depicted in Figure 4A, unveil that the sole overexpression of miR-21 induces both drug resistance and EMT. Conversely, as evidenced in Figure 4B, the combination of miR-21 knockdown with PTEN overexpression yields notable outcomes: cell cycle arrest at 17%, senescence at 20%, autophagy at 26%, and apoptosis at 37%. Upon simultaneous overexpression of both PTENP1 and PTEN alongside miR-21 knockdown, a distinct pattern emerges with cell cycle arrest observed at 16%, senescence at 24%, autophagy at 28%, and apoptosis at 32% (Figure 4C). Notably, overexpressing PTENP1 alone yields a distinct distribution with 3% cell cycle arrest, 15% senescence, 7% autophagy, and 75% apoptosis (Figure 4D).

Our findings strongly support a combined approach targeting drug resistance and EMT through miR-21 knockdown alongside PTEN and/or PTENP1 overexpression. As illustrated in Figure 4B,C, this strategy enhances autophagic and apoptotic cell death. Moreover, PTENP1 overexpression (Figure 4D) emerges as a promising therapeutic avenue, promoting increased apoptosis. By integrating miR-21 knockdown with PTEN and/or PTENP1 overexpression, we aim to effectively address drug resistance and EMT, thereby significantly improving cancer therapy outcomes. For more details, refer to Figure 4.

### 2.4. Mapping Molecular Circuits: Patterns and Dynamics

Gene regulatory networks (GRNs) are pivotal for deciphering the dynamics of biological systems, and it is essential to understand the positive and negative circuits that form them. Our recent analysis aimed to validate our network’s ability to mirror these circuits, resulting in the identification of 39 biological circuits that significantly influence network dynamics. Among the thirty-nine identified circuits, our focus narrowed to twenty-two specific ones, each involving up to three molecular elements (detailed in Table S2). Many of these circuits have already undergone experimental verification, as outlined in Table S2. Intriguingly, among these circuits, we identified eight novel positive circuits and one negative circuit that remain unexplored experimentally. These are detailed in Table 2, underscoring the need for further investigation. While the exact roles of biochemical interactions within these circuits in the intra-S/G2-M checkpoint mechanism of cancer cells are still unclear, their interactions are well documented in the literature, as summarized in Table S3.

This knowledge gap served as the impetus for conducting perturbation experiments, which were aimed at elucidating the significance of these circuits in modulating cellular phenotypes, particularly in the context of EMT and drug resistance. Table 3 provides concise insights into each circuit’s perturbations and resulting outcomes, with specific emphasis on outcomes highlighted in bold within Table S4. Across various positive circuits involving key regulatory elements, like PTEN, PTENP1, BMI1, AKT, YY1, NFkB, SNAIL, and ATM, perturbations result in a spectrum of phenotypic outcomes, including drug resistance, EMT, cell cycle arrest, senescence, autophagy, and apoptosis, as demonstrated in Table 3. Interestingly, the perturbation of different nodes within these circuits leads to nuanced alterations in cellular behavior, highlighting the intricate interplay between molecular components. For additional information, refer to Table S4. These findings underscore the intricate and multifaceted nature of cellular responses to perturbations.

In addition, it is important to note that during the perturbation analysis of the PTEN/BMI1/ATM and PTEN/Cdc25/ATM positive circuits, as well as the PTEN/E2F1/ATM negative circuit, oscillations were detected in only a limited number of cases. These instances were associated with cyclic attractors, suggesting the possibility that cells are evading cycle arrest, as proposed in prior research by Reyes et al. [31] and Sarin et al. [32]. Consequently, we have documented these findings in Table S5 and opted not to delve further into these occurrences. 

Our comprehensive analysis of cellular circuits has unveiled eight groundbreaking positive circuits and one negative circuit intricately associated with drug resistance and EMT. Central to these circuits lies PTEN, intricately interacting with key transcription factors and proteins like PTENP1, AKT, E2F1, BMI1, Cdc25, and NFkB. Our profound findings emphasize the paramount pursuit of exploring these intricate regulatory networks involving PTEN and its counterparts, opening avenues for promising strategies against drug resistance and EMT in cancer. 

### 2.5. Network-Based Therapeutic Strategies for Combating Drug Resistance and EMT

**Figure 5:**
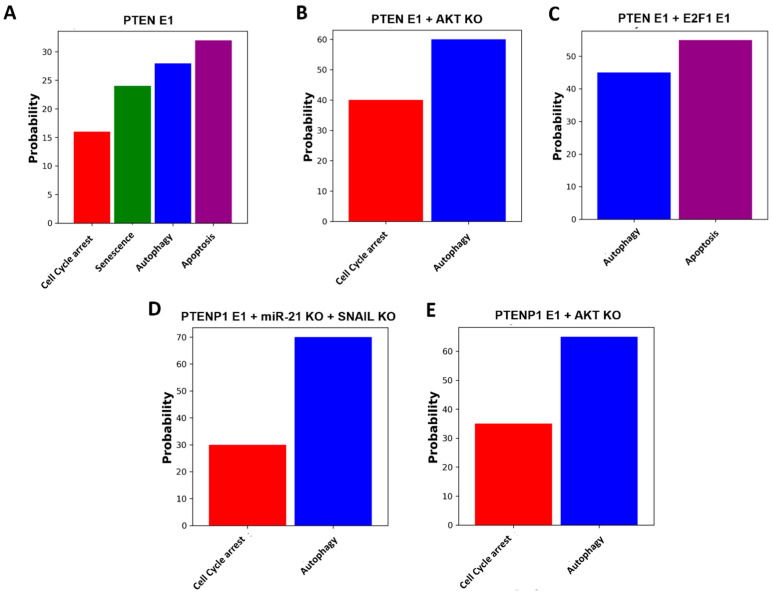
Network-based strategies for combating drug resistance and EMT: experimental perturbations and simulation outcomes. Overexpression (E1) represents gain of function (GoF) and knockdown (KO) represents loss of function (LoF) perturbations. (**A**) Overexpression (E1) of PTEN. (**B**) Overexpression (E1) of PTEN with knockdown (KO) of AKT. (**C**) Overexpression (E1) of PTEN alongside overexpression (E1) of E2F1. (**D**) Overexpression (E1) of PTENP1 with knockdown (KO) of miR-21 and SNAIL. (**E**) Overexpression (E1) of PTENP1 with knockdown (KO) of AKT. Each bar represents its corresponding phenotype. Extensive Monte Carlo simulations (100,000 iterations) were conducted for each perturbation to ensure robustness and reliability. For more details, see Section 2.5.

In our study, we propose experimental designs based on dynamic Boolean networks to explore the modulatory effects of PTEN/miR-21 and PTENP1 on drug resistance and EMT, alongside their broader impact on cell fate decisions. Inspired by methodologies that integrate computational predictions with experimental validation, our approach aims to bridge the gap between theoretical models and clinical realities. We propose five specific perturbations in response to DNA damage. Additionally, we conducted Monte Carlo simulations with 100,000 runs for each case, as depicted in Figure 5.

In the first perturbation (Figure 5A), amplifying PTEN expression triggers a cascade of cellular responses: halting the cell cycle at 16%, entering senescence at 24%, inducing autophagy at 28%, and initiating apoptosis at 32%. Moving to the second perturbation (Figure 5B), AKT knockdown combined with PTEN overexpression results in 40% cell cycle arrest and 60% autophagy. In the third perturbation (Figure 5C), PTEN overexpression alongside E2F1 overexpression leads to 45% autophagy and 55% apoptotic cell death. Next, the fourth perturbation (Figure 5D) involves PTENP1 overexpression coupled with miR-21 and SNAIL knockdown, resulting in 30% cell cycle arrest and 70% autophagy. Lastly, the fifth perturbation (Figure 5E) entails PTENP1 overexpression and AKT knockdown, yielding 35% cell cycle arrest and 65% autophagy, serving as potential strategies for inhibiting EMT and drug resistance.

These predictions open avenues for in vivo and in vitro studies to validate the network’s hypotheses and identify novel therapeutic targets and strategies in cancer treatment.

## 3. Discussion

In this study, we introduce a groundbreaking dynamic Boolean network model that sheds light on the complex regulatory interactions among PTENP1, miR-21, and PTEN, as shown in Figure 1. Our model represents a pioneering effort in the field, providing a comprehensive framework to elucidate the intricate signaling pathways involved in EMT, drug resistance, and crucial cell fate decisions, such as cell cycle arrest, senescence, autophagy, and apoptosis.

Our model also provides insights into the dynamic nature of cellular decision-making processes. Our research has unveiled six distinct cellular states, or fixed points, each characterized by its unique molecular mechanisms and outcomes, as illustrated in Figure 2A. These fixed points encompass drug resistance, cell cycle arrest, senescence, autophagy, apoptosis, and EMT. Understanding these underlying mechanisms is pivotal for devising targeted therapeutic interventions aimed at overcoming drug resistance in cancer treatment. Specifically, our findings are particularly relevant to breast cancer, hepatocellular carcinoma (HCC), and oral squamous cell carcinoma (OSCC), where the miR-21/PTEN axis plays a critical role in modulating cellular behaviors.

Our model thoroughly analyzes molecular mechanisms governing diverse cellular states. Key fixed points include cell cycle arrest and senescence driven by p53-A activation and p21 induction. Additionally, pathways like autophagy (ULK1) and apoptosis (p53-K, BAX, Caspase-3) emerge. Drug resistance and EMT are facilitated by miR-21, BCL2, NFkB, SNAIL, ZEB1, and VIM (see Figure 2A). Employing non-deterministic network dynamics, we assign probabilities to fixed points, revealing drug resistance and EMT prevalences of 50% under “neutral” conditions (input OFF, decreasing to 46% ON). Conversely, cell cycle arrest, senescence, apoptosis, and autophagy each manifest at 1%, indicating the robust occurrence of drug resistance and EMT, regardless of DNA damage (Figure 2B).

Additionally, we validated the model using gain of function (GOF) and loss of function (LOF) perturbations (Figure 3), confirming its predictive capability. In breast cancer, our model aligns with studies showing miR-21’s inhibition of PTEN, promoting EMT, drug resistance, and proliferation [21,24]. Conversely, PTEN upregulation via small molecules or PTENP1 overexpression inhibits EMT and drug resistance, activating cell cycle arrest and apoptosis pathways [19,24]. Similarly, in HCC, our model mirrors miR-21 inhibition, leading to G2/M cell cycle arrest and apoptosis and hindering cancer cell proliferation and migration [28]. Furthermore, in OSCC, our model highlights the PTEN/miR-21 and PTENP1 axis in proliferation, invasion, and cell cycle modulation, underscoring its clinical relevance in differentiation and prognosis [9]. Our research underscores the effectiveness of our dynamic model in unraveling clinically relevant mechanisms driving cancer development and progression. 

Moreover, our investigation assessed the impact of PTEN/miR-21 and PTENP1 on drug resistance and EMT, influencing cell fate decisions in DDR. We examined four distinct perturbations: overexpressing miR-21, knocking down miR-21 while overexpressing PTEN, knocking down miR-21 while overexpressing both PTEN and PTENP1, and overexpressing PTENP1 alone (see Figure 4). Our Monte Carlo simulations, involving 100,000 runs for each perturbation, revealed that the sole overexpression of miR-21 induces drug resistance and EMT. Knocking down miR-21 with PTEN overexpression led to cell cycle arrest (17%), senescence (20%), autophagy (26%), and apoptosis (37%). Simultaneous overexpression of PTENP1 and PTEN with miR-21 knockdown showed cell cycle arrest (16%), senescence (24%), autophagy (28%), and apoptosis (32%). Overexpressing PTENP1 alone resulted in 3% cell cycle arrest, 15% senescence, 7% autophagy, and 75% apoptosis. These findings indicate that a combined approach targeting miR-21, PTEN, and PTENP1 effectively addresses drug resistance and EMT, enhancing autophagic and apoptotic cell death.

Furthermore, biochemical circuits are pivotal in GRNs, encompassing positive and negative interactions crucial for understanding biological system dynamics [33]. Our study identified eight novel positive circuits and one negative circuit (e.g., PTEN/PTENP1/miR-21, PTEN/E2F1/miR-21), which are detailed in Table S3. Perturbation analyses of these circuits (see Table 3 and Table S4 for more details) revealed that the overexpression of miR-21, SNAIL, YY1, NFkB, and PTEN induces EMT and drug resistance, alongside other cellular responses such as cell cycle arrest, senescence, apoptosis, and autophagy. This multifaceted manifestation underscores the complexity inherent in cellular responses to external stimuli. Our findings indicate that such perturbations, either individually or in combination, consistently induce EMT and confer resistance to therapeutic agents. These molecules are well-established regulators of EMT and have been implicated in promoting drug resistance in various cellular contexts, as shown in the recently reviewed by Dillen et al. [34]. 

Additionally, our analysis has revealed perturbations involving ATM, a crucial regulator of DNA damage response and repair, demonstrating associations with both the induction of EMT and drug resistance (see Table S4, denoted by bold fonts). One plausible explanation for this phenomenon lies in the intricate interplay between ATM-mediated DNA damage signaling pathways and cellular processes governing EMT and drug resistance.

Emerging evidence suggests that ATM activation can promote EMT through various mechanisms, including the regulation of transcription factors, such as SNAIL and VIM, well-known drivers of EMT programs. Notably, a previous in vivo/in vitro study [35] elucidated the significant contribution of the ATM-SNAIL pathway to EMT induction across diverse cell lines, including HeLa, MCF-7, HEK293, and MDA-MB-231, particularly under Camptothecin (CPT) treatment and ionizing irradiation (IR). Recent investigations in HCT-116 and A549 cell lines further corroborate these findings [36], highlighting ATM’s pivotal role in facilitating SNAIL/VIM functions, inhibiting apoptosis, and amplifying EMT and drug resistance in response to CPT treatment. This suggests that ATM’s functionality not only fosters EMT but also confers resistance to chemotherapy drugs, such as CPT or IR. In this context, our findings suggest that the co-activation of PTEN alongside ATM serves to suppress EMT and counteract drug resistance by influencing key cellular fate determinants, such as apoptosis and autophagy. For more details, see Table S4. Our findings underscore the complexity of cellular responses to stimuli and highlight their roles in cancer drug resistance. 

In addition, based on our model, we proposed five testable predictions, rigorously validated through Monte Carlo simulations (100,000 runs, depicted in Figure 5). These predictions provide crucial insights for future experimental validations and the development of novel therapeutic strategies in cancer management. Despite significant strides in understanding regulatory circuits, several areas remain unexplored. Our findings indicate that these newly discovered circuits could profoundly impact drug resistance and EMT in cancer cells. Future studies should focus on rigorous experimental validation, both in vitro and in vivo, to confirm these circuits’ relevance to drug resistance and EMT. Moreover, exploring interactions between these circuits and established pathways could enhance our understanding of the regulatory network governing drug resistance in cancer cells. However, our methodology has limitations, particularly in predicting expression levels and time-dependent dynamics.

## 4. Materials and Methods

### 4.1. Mapping the Gene Regulatory Network Landscape in Cancer Cells: A Fusion of Public Databases and Tools

We devised a comprehensive gene regulatory framework to investigate the functions of PTENP1 and miR-21 ncRNAs through detailed exploration using PubMed and BIOGRID 3.5 (https://thebiogrid.org/ accessed on 22 July 2024) [37] and employed GINsim 3.0.0b (http://www.ginsim.org/downloads accessed on 22 July 2024) [38] to construct, simulate, and visualize outcomes within a Boolean model. GINsim, accessible through academic channels, adeptly identifies all attractors within wild-type and mutant systems. For accessibility to the model file, see the “Data Availability” section.

### 4.2. Developing Dynamic Boolean Network Models, Rules, and Simulations from PubMed-Based Insights

The Boolean methodology involves analyzing a regulatory graph where nodes represent signaling components and edges signify activation or inhibition. Each node is a Boolean variable, either “0” (inactive) or “1” (active). Logical rules, derived from biochemical data, determine node activation [39]. The interactions within the gene regulatory network of PTENP1 and miR-21 were translated into Boolean rules based on the biological literature (Supplementary Table S1). Using “AND,” “OR,” and “NOT” operators, these rules were crafted. Simulations yield attractors, and a state transition graph (STG) helps understand the model’s dynamics. Each STG node represents the network’s current state, with arcs showing state transitions. Stable states lack outgoing edges, while cyclic states have confined transitions. Asynchronous updates reflect the non-deterministic nature of molecular networks [40,41,42,43,44,45,46,47,48,49,50]. This approach allows for in silico perturbations, such as gain of function (GoF) or loss of function (LoF), manipulating node values to examine their effects on network dynamics and resulting phenotypes. Additionally, negative and positive circuits guide network dynamics, with negative circuits potentially inducing oscillations and positive circuits governing multi-stable dynamics. Such methodologies facilitate exploring the effects of nodes on network dynamics and subsequent phenotypic outcomes, supporting the investigation of specific node influences on network dynamics and resulting phenotypes [38,39].

## 5. Conclusions

**Figure 6:**
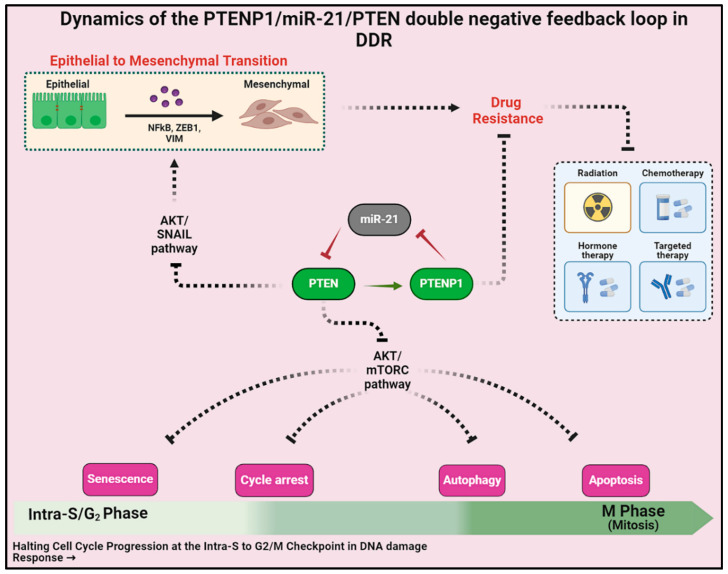
The lncRNA PTENP1/miR-21/PTEN axis in DDR: A double negative feedback loop combating drug resistance and EMT. This intricate interplay orchestrates vital cellular processes, inhibiting the AKT/SNAIL pathway to halt EMT progression and fortify drug resistance. PTENP1 upregulation suppresses miR-21, boosting PTEN levels. PTEN’s elevation impedes the AKT/mTOR pathway, regulating cell cycle, senescence, autophagy, and apoptosis. This axis emerges as a pivotal target for novel cancer therapies.

In summary, our research delves into the mechanisms of drug resistance and EMT in cancer cells, exploring the intricate interplay among PTENP1/miR-21/PTEN. Our findings offer new insights into understanding these malignancies and suggest a potential avenue for treatment. By inhibiting miR-21, we can bolster PTEN levels and subsequently impede drug resistance and EMT in cancer cells. This is particularly promising as our investigation has demonstrated that PTEN is effective in suppressing EMT and drug resistance by targeting the AKT/SNAIL and AKT/mTOR pathways, as depicted in Figure 6. Our findings represent a potential breakthrough in the fight against cancer and provide a stepping stone for further research in this domain.

## Figures and Tables

**Table 2:** Predictive feedback loops: The Boolean network identified eight positive feedback loops and one negative feedback loop.

Predictive Feedback Loops
Positive feedback loops
PTEN/PTENP1/miR-21
PTEN/E2F1/miR-21
PTEN/BMI1/ATM
PTEN/Cdc25/ATM
PTEN/AKT/SNAIL
PTEN/PTENP1/YY1
PTEN/NFkB/YY1
PTEN/NFkB/SNAIL
Negative feedback loop
PTEN/E2F1/ATM

**Table 3:** Comprehensive perturbation analysis: positive and negative circuits overview.

Positive Circuits	Perturbations	Phenotypes
PTEN/PTENP1/miR-21	Various	Drug Resistance, EMT, Cell Cycle Arrest, Senescence, Autophagy, Apoptosis.
PTEN/E2F1/miR-21	Various	Drug Resistance, EMT, Cell Cycle Arrest, Senescence, Autophagy, Apoptosis.
PTEN/BMI1/ATM	Various	EMT, Senescence, Drug Resistance, Cell Cycle Arrest, Autophagy, Apoptosis.
PTEN/Cdc25/ATM	Various	EMT, Senescence, Drug Resistance, Cell Cycle Arrest, Autophagy, Apoptosis.
PTEN/AKT/SNAIL	Various	Drug Resistance, EMT, Cell Cycle Arrest, Senescence, Autophagy, Apoptosis.
PTEN/PTENP1/YY1	Various	Drug Resistance, EMT, Cell Cycle Arrest, Senescence, Autophagy, Apoptosis.
PTEN/NFkB/YY1	Various	Drug Resistance, EMT, Cell Cycle Arrest, Senescence, Autophagy, Apoptosis.
PTEN/NFkB/SNAIL	Various	Drug Resistance, EMT, Cell Cycle Arrest, Senescence, Autophagy, Apoptosis.
Negative Circuit	Perturbations	Phenotypes
PTEN/E2F1/ATM	Various	Drug Resistance, EMT, Cell Cycle Arrest, Senescence, Autophagy, Apoptosis.

## Data Availability

The model code is available in a publicly accessible GitHub repository, which can be accessed through the following link: https://github.com/GuptaShan/Dynamic-PTENP1-miR-21-PTEN-BNM-Model.git (accessed on 22 July 2024).

## Supplementary Materials

The following supporting information can be downloaded at https://www.mdpi.com/article/10.3390/ijms25158264/s1. References [51,52,53,54,55,56,57,58,59,60,61,62,63,64,65,66,67,68,69,70,71,72,73,74,75,76,77,78,79,80,81,82,83,84,85] are cited in the supplementary materials.
