# Supplementary material for: LncRNA PTENP1/miR-21/PTEN Axis Modulates EMT and Drug Resistance in Cancer: Dynamic Boolean Modeling for Cell Fates in DNA Damage Response
Source: Int J Mol Sci. 2024 Jul 29;25(15):8264. doi: 10.3390/ijms25158264 (PMC11311614; doi:10.3390/ijms25158264)
Supplement: Supplementary file 1 [file ijms-25-08264-s001.zip › ijms-3117243-supplementary/File S1.pdf]

File S1

Deciphering DNA Damage Mechanisms in EMT, Drug Resistance, and Cell Fate Decisions at the Intra-S to G2/M Checkpoint

Extensive research has been dedicated to unraveling the intricate response of DNA damage repair (DDR) pathways to stress, particularly their impact on cell cycle progression [1]. Activation of the G1/S or G2/M phases within the DDR, induced by DNA damage, results in subsequent cycle arrest or senescence, facilitating repair mechanisms such as autophagy or apoptosis [1,2]. At the core of this response lies the activation of the ATM kinase by DNA double-strand breaks (DSBs), which regulates downstream effectors like p53, initiating a cascade of events culminating in cell cycle arrest [3].

Our model, inspired by the diverse phosphorylation modes of p53 described by Zhang et al. [4], emphasizes additional nodes in the signaling pathway. Specifically, we highlight the significance of the p53-associated E3 ubiquitin-protein ligase Mdm2 (Mdm2) interactions [5], which necessitates the activation of p53-Arrest (p53-A) and p53-Killer (p53-K). In our model, p53-A corresponds to phosphorylation at Ser-15 and Ser-20, while p53-K signifies heightened phosphorylation at Ser-46. These phosphorylation events are crucial for regulating processes such as cell cycle arrest, senescence or autophagic and apoptotic cell deaths. P21, Protein phosphatase 1D (Wip1), and tumor protein p53-inducible nuclear protein 1 (TP53INP1) are triggered by p53-A. Similarly, p53-K directly inhibits Apoptosis regulator Bcl-2 (Bcl2) while positively interacting with apoptosis inducers like Apoptosis regulator BAX (BAX) and Caspase-3 (caspase-3) in cancer cells [6], subsequently activating PTEN to suppress the AKT/mTOR signaling pathway [7]. In addition, PTEN activation is also triggered by p53-K [8].

Interestingly, miR-21 expression is directly induced by AKT and/or E2F1 [9,10], both of which directly target PTEN expression and activate the AKT/mTOR pathway. In addition, E2F1 also activates Myc proto-oncogene protein (Myc) and Sirtuin 1 (Sirt1) [11,12]**.** Moreover, PTEN induces PTENP1 expression [13], which subsequently inhibits miR-21 activity [13]. Our study investigates the pivotal role of the PTENP1/miR-21 and PTEN axis in triggering the intra-S to G2/M-phase transition in cancer cells. PTENP1 directly inhibits CDK2-Cyclin A2, thereby halting the cell cycle at the intra-S to G2/M checkpoint [14]. Additionally, p21 directly suppresses the CDK2/Cyclin A2 and

CDK1/Cyclin B complex, inducing arrest at the intra-S to G2/M phase [15], with AKT serving as a negative regulator of p21 activity [16]. Additionally, the interplay between Caspase3 and p21 highlights the delicate balance between cell survival and death, as p21 serves as a regulator of cell cycle arrest and apoptosis, while Caspase3 is a key executioner of apoptosis [17].

Moreover, the integration of closed pathways, such as Raf kinase inhibitor protein (RKIP)/Nuclear factor NF-kappa-B p105 subunit (NFkB)/ Zinc finger protein SNAI1 (SNAIL), unveils the intricate interplay among signaling pathways implicated in metastasis and invasion [18]. Similarly, the dynamic balance within the closed pathway of Myc and p21 elucidates the complex regulatory mechanisms governing cell cycle progression [19]. Additionally, the interconnectedness observed in the closed pathway of AKT and mammalian target of rapamycin complex 2 (mTORC2) sheds light on a critical signaling cascade regulating cell survival and metabolism [20]. The integration of Cdc25 and ATM within a closed pathway suggests insights into ATM's pivotal role in modulating cell cycle checkpoints by regulating Cdc25 activity [21]. Furthermore, the coordinated regulation within the closed pathway of Serine/threonine-protein kinase ULK1 (ULK1) and mammalian target of rapamycin complex 1 (mTOR1) underscores the intricate orchestration of autophagy [22]. Lastly, the interplay among PTEN, AKT, and miR-21 within a closed pathway delineates a critical signaling axis governing cell proliferation, survival, and invasion. While PTEN exerts tumor-suppressive effects, AKT and miR-21 promote oncogenic signaling, contributing to cancer progression [9].

Moreover, ATM positively regulates PTEN expression [23], inducing autophagy through the PTEN and AMP-activated protein kinase (AMPK) pathways, which in turn inhibit mTOR 1/2, activating the ULK1/Beclin 1 complex, thereby making PTEN-inducible autophagy ATM/AMPK pathway-dependent [23]. Furthermore, the closed pathway between E2F1 and ATM [3] indicates that ATM plays a crucial role in regulating both cell cycle progression and DNA repair, particularly in response to DNA damage [3]. Furthermore, NF-κB orchestrates EMT and confers drug resistance by activating transcription factors SNAIL and Yin Yang 1 (YY1) [24]. These factors drive cellular programs promoting the transition of cells from an epithelial to a mesenchymal phenotype, enhancing migratory and invasive capabilities. NF-κB-mediated activation of SNAIL and YY1 also contributes to drug resistance acquisition, complicating treatment strategies in diseases like cancer [25,26]. In this context, upregulated PTEN inhibits

AKT/SNAIL pathway [27,28], thereby blocking EMT and drug resistance and offering potential therapeutic avenues.

Drawing from the foundational interactions outlined earlier, we constructed our Boolean model to elucidate the regulation of cell death pathways in cancer cells.

References:

1. Barnum, K.J.; O'Connell, M.J. Cell Cycle Regulation by Checkpoints. *Methods Mol Biol* **2014**, *1170*, 29–40, doi:10.1007/978-1-4939-0888-2_2.
2. Bertoli, C.; Skotheim, J.M.; de Bruin, R.A.M. Control of Cell Cycle Transcription during G1 and S Phases. *Nat Rev Mol Cell Biol* **2013**, *14*, 518–528, doi:10.1038/nrm3629.
3. Lin, W.C.; Lin, F.T.; Nevins, J.R. Selective Induction of E2F1 in Response to DNA Damage, Mediated by ATM-Dependent Phosphorylation. *Genes Dev* **2001**, *15*, 1833–1844.
4. Zhang, X.-P.; Liu, F.; Wang, W. Two-Phase Dynamics of P53 in the DNA Damage Response. *Proc Natl Acad Sci U S A* **2011**, *108*, 8990–8995, doi:10.1073/pnas.1100600108.
5. Lev Bar-Or, R.; Maya, R.; Segel, L.A.; Alon, U.; Levine, A.J.; Oren, M. Generation of Oscillations by the P53-Mdm2 Feedback Loop: A Theoretical and Experimental Study. *Proceedings of the National Academy of Sciences* **2000**, *97*, 11250–11255.
6. Shen, Y.; White, E. P53-Dependent Apoptosis Pathways. *Adv Cancer Res* **2001**, *82*, 55–84, doi:10.1016/s0065-230x(01)82002-9.
7. Wang, H.; Fan, Q.; Zhang, L.; Shi, D.; Wang, H.; Wang, S.; Bian, B. Folate-Targeted PTEN/AKT/P53 Signaling Pathway Promotes Apoptosis in Breast Cancer Cells. *Pteridines* **2020**, *31*, 158–164, doi:10.1515/pteridines-2020-0020.
8. Stambolic, V.; MacPherson, D.; Sas, D.; Lin, Y.; Snow, B.; Jang, Y.; Benchimol, S.; Mak, T.W. Regulation of PTEN Transcription by P53. *Mol Cell* **2001**, *8*, 317–325, doi:10.1016/s1097-2765(01)00323-9.
9. Sayed, D.; Abdellatif, M. AKT-Ing via microRNA. *Cell Cycle* **2010**, *9*, 3233–3237, doi:10.4161/cc.9.16.12634.
10. Qin, S.; Xu, J.; Yi, Y.; Jiang, S.; Jin, P.; Xia, X.; Ma, F. Transcription Factors and Methylation Drive Prognostic miRNA Dysregulation in Hepatocellular Carcinoma. *Front. Oncol.* **2021**, *11*, doi:10.3389/fonc.2021.691115.
11. Coller, H.A.; Forman, J.J.; Legesse-Miller, A. "Myc'ed Messages": Myc Induces Transcription of E2F1 While Inhibiting Its Translation via a microRNA Polycistron. *PLoS Genet* **2007**, *3*, e146, doi:10.1371/journal.pgen.0030146.
12. Wang, C.; Chen, L.; Hou, X.; Li, Z.; Kabra, N.; Ma, Y.; Nemoto, S.; Finkel, T.; Gu, W.; Cress, W.D.; et al. Interactions between E2F1 and SirT1 Regulate Apoptotic Response to DNA Damage. *Nat Cell Biol* **2006**, *8*, 1025–1031, doi:10.1038/ncb1468.
13. Yu, G.; Yao, W.; Gumireddy, K.; Li, A.; Wang, J.; Xiao, W.; Chen, K.; Xiao, H.; Li, H.; Tang, K.; et al. Pseudogene PTENP1 Functions as a Competing Endogenous RNA to Suppress Clear-Cell Renal Cell Carcinoma Progression. *Molecular Cancer Therapeutics* **2014**, *13*, 3086–3097, doi:10.1158/1535-7163.MCT-14-0245.
14. Chen, S.; Wang, Y.; Zhang, J.-H.; Xia, Q.-J.; Sun, Q.; Li, Z.-K.; Zhang, J.-G.; Tang, M.-S.; Dong, M.-S. Long Non-Coding RNA PTENP1 Inhibits Proliferation

and Migration of Breast Cancer Cells via AKT and MAPK Signaling Pathways. *Oncology Letters* **2017**, *14*, 4659, doi:10.3892/ol.2017.6823.
15. Bačević, K.; Lossaint, G.; Achour, T.N.; Georget, V.; Fisher, D.; Dulić, V. Cdk2 Strengthens the Intra-S Checkpoint and Counteracts Cell Cycle Exit Induced by DNA Damage. *Sci Rep* **2017**, *7*, 13429, doi:10.1038/s41598-017-12868-5.
16. Park, J.K.; Jung, H.-Y.; Park, S.H.; Kang, S.Y.; Yi, M.-R.; Um, H.D.; Hong, S.H. Combination of PTEN and γ-Ionizing Radiation Enhances Cell Death and G2/M Arrest Through Regulation of AKT Activity and P21 Induction in Non–Small-Cell Lung Cancer Cells. *International Journal of Radiation Oncology*Biology*Physics* **2008**, *70*, 1552–1560, doi:10.1016/j.ijrobp.2007.11.069.
17. Zhang, Y.; Fujita, N.; Tsuruo, T. Caspase-Mediated Cleavage of p21Waf1/Cip1 Converts Cancer Cells from Growth Arrest to Undergoing Apoptosis. *Oncogene* **1999**, *18*, 1131–1138, doi:10.1038/sj.onc.1202426.
18. Lin, K.; Baritaki, S.; Militello, L.; Malaponte, G.; Bevelacqua, Y.; Bonavida, B. The Role of B-RAF Mutations in Melanoma and the Induction of EMT via Dysregulation of the NF-κB/Snail/RKIP/PTEN Circuit. *Genes & Cancer* **2010**, *1*, 409–420, doi:10.1177/1947601910373795.
19. Jänicke, R.U.; Sohn, D.; Essmann, F.; Schulze-Osthoff, K. The Multiple Battles Fought by Anti-Apoptotic P21. *Cell Cycle* **2007**, *6*, 407–413, doi:10.4161/cc.6.4.3855.
20. Yang, G.; Murashige, D.S.; Humphrey, S.J.; James, D.E. A Positive Feedback Loop between Akt and mTORC2 via SIN1 Phosphorylation. *Cell reports* **2015**, *12*, 937–943.
21. Agarwal, C.; Tyagi, A.; Agarwal, R. Gallic Acid Causes Inactivating Phosphorylation of cdc25A/cdc25C-Cdc2 via ATM-Chk2 Activation, Leading to Cell Cycle Arrest, and Induces Apoptosis in Human Prostate Carcinoma DU145 Cells. *Mol Cancer Ther* **2006**, *5*, 3294–3302, doi:10.1158/1535-7163.MCT-06-0483.
22. Holczer, M.; Hajdú, B.; Lőrincz, T.; Szarka, A.; Bánhegyi, G.; Kapuy, O. A Double Negative Feedback Loop between mTORC1 and AMPK Kinases Guarantees Precise Autophagy Induction upon Cellular Stress. *International Journal of Molecular Sciences* **2019**, *20*, 5543, doi:10.3390/ijms20225543.
23. Chen, J.-H.; Zhang, P.; Chen, W.-D.; Li, D.-D.; Wu, X.-Q.; Deng, R.; Jiao, L.; Li, X.; Ji, J.; Feng, G.-K.; et al. ATM-Mediated PTEN Phosphorylation Promotes PTEN Nuclear Translocation and Autophagy in Response to DNA-Damaging Agents in Cancer Cells. *Autophagy* **2015**, *11*, 239–252, doi:10.1080/15548627.2015.1009767.
24. Pires, B.R.B.; Mencalha, A.L.; Ferreira, G.M.; de Souza, W.F.; Morgado-Díaz, J.A.; Maia, A.M.; Corrêa, S.; Abdelhay, E.S.F.W. NF-kappaB Is Involved in the Regulation of EMT Genes in Breast Cancer Cells. *PLoS One* **2017**, *12*, e0169622, doi:10.1371/journal.pone.0169622.
25. Hu, Z.; Liu, X.; Tang, Z.; Zhou, Y.; Qiao, L. Possible Regulatory Role of Snail in NF-κB-Mediated Changes in E-Cadherin in Gastric Cancer. *Oncology Reports* **2013**, *29*, 993–1000, doi:10.3892/or.2012.2200.
26. Dillen, A.; Bui, I.; Jung, M.; Agioti, S.; Zaravinos, A.; Bonavida, B. Regulation of PD-L1 Expression by YY1 in Cancer: Therapeutic Efficacy of Targeting YY1. *Cancers* **2024**, *16*, 1237, doi:10.3390/cancers16061237.
27. Prasad, P.; Vasas, A.; Hohmann, J.; Bishayee, A.; Sinha, D. Cirsiliol Suppressed Epithelial to Mesenchymal Transition in B16F10 Malignant Melanoma Cells

through Alteration of the PI3K/Akt/NF-κB Signaling Pathway. *International Journal of Molecular Sciences* **2019**, *20*, 608, doi:10.3390/ijms20030608.
28. He, E.; Pan, F.; Li, G.; Li, J. Fractionated Ionizing Radiation Promotes Epithelial-Mesenchymal Transition in Human Esophageal Cancer Cells through PTEN Deficiency-Mediated Akt Activation. *PLOS ONE* **2015**, *10*, e0126149, doi:10.1371/journal.pone.0126149.
