# Supplementary material for: LncRNA PTENP1/miR-21/PTEN Axis Modulates EMT and Drug Resistance in Cancer: Dynamic Boolean Modeling for Cell Fates in DNA Damage Response
Source: Int J Mol Sci. 2024 Jul 29;25(15):8264. doi: 10.3390/ijms25158264 (PMC11311614; doi:10.3390/ijms25158264)
Supplement: Supplementary file 1 [file ijms-25-08264-s001.zip › ijms-3117243-supplementary/Table S1.pdf]

Table S1

Table S1: Logical Rules Governing Node States in the Model (Highlighted in Blue) Illustrated in Fig. 1 (main paper), Depicting the State of Each Node Based on Its Regulators. The left-hand side presents the official names of the molecules (target nodes) (highlighted in cream colour), while the right-hand side lists the references of each interaction leading to the target node.

Official names of the molecules	Target Node	No. Interactions	Interaction	Biological Justification	References
ATM serine/threonine kinase	ATM	1	DNA damage	DNA damage triggers ATM activity.	PMID: 15322239
		2	Wip1	Wip1 inhibits ATM activity.	PMID: 22768840
		3	E2F1	E2F1 induces ATM expression.	PMID: 11459832
		4	BMI1	BMI1 is a negative regulator of ATM expression.	PMID: 26425649
		5	Cdc25	Cdc25 is a negative regulator of ATM expression.	PMID: 22986406
		Rule	DNA_Damage AND (NOT Wip1 OR E2F1 OR NOT (BMI1 OR Cdc25))		ATM can be activated when DNA damage is present and Wip is absent, or when E2F1 is present, or when BMI1 is absent, or when Cdc25 is absent.
AMP-activated protein kinase	AMPK	6	ATM	ATM triggers activation of AMPK.	PMID: 28964605
		7	Wip1	Wip1 inhibits MAPK activity.	PMID: 37876819
		Rule	ATM AND NOT Wip1		AMPK can be activated when ATM is present and Wip1 is absent.
		8	Wip1	Wip1 inhibits Mdm2 expression.	PMID: 17936559
		9	p53	p53 induces Mdm2 expression.	PMID: 12563309
		10	ATM	ATM is negative regulator of Mdm2 activity.	PMID: 16082221

E3 ubiquitin protein ligase homolog protein	Mdm2	11	PTEN	PTEN stabilized p53 by inhibits Mdm2 expression.	PMID: 11729185
		12	AKT	AKT is a positive regulator of Mdm2 activity.	PMID: 11923280
		Rule	(NOT Wip1 OR p53) AND NOT ATM AND NOT PTEN AND AKT		Mdm2 can be activated when Wip1 is absent or p53 is present and ATM is absent and PTEN is absent and AKT is present.
Tumor supressor p53 protein	p53	13	ATM	ATM is positive regulator of p53 activity.	PMID: 11526498
		14	Mdm2	Mdm2 is negative regulator of p53 activity.	PMID: 12563309
		15	AMPK	AMPK can induces p53 expression.	PMID: 15866171
		16	YY1	YY1 is a negative regulator of p53 activity.	PMID: 15295102
		Rule	ATM OR (NOT Mdm2 AND AMPK AND NOT YY1)		p53 can be activated when ATM is present or Mdm2 is absent and AMPK is present and YY1 is absent.
Tumor supressor p53 protein (Ser-15 and Ser-20)	p53-A	17	Sirt1	Sirt1 is a negative regulator of p53-A activity.	PMID: 20471503
		18	p53-K	p53-K directly inhibits p53-A.	PMID: 21576488
		19	p53	p53-A induces by p53.	PMID: 21576488
		20	p53INP1	p53INP1 inhibits p53-A.	PMID: 21576488
		Rule	NOT Sirt1 AND NOT p53-K AND (p53 OR NOT p53INP1)		p53_A can be activated in the absence of Sirt1 and the absence of p53_K and the presence of p53 or the absence of p53_INP1.
Tumor protein p53 inducible nuclear protein 1	p53-INP1	21	p53-k	p53-K induces p53INP1 expression.	PMID: 21576488
		22	p53-A	p53-A positively regulate p53INP1 expression.	PMID: 21576488
		Rule	p53-K OR p53-A		p53INP1 expression induced by p53-A or p53-K.

Tumor supressor p53 protein (Ser-46)	p53-K	23	p53-A	p53-A inhibits p53-K expression.	PMID: 21576488
		24	Sirt1	Sirt1 is a negative regulator of p53-K activity.	PMID: 20471503
		25	Wip1	Wip1 is a negative regulator of p53-K activity.	PMID: 27959454
		26	p53	p53 induces p53-K expression.	PMID: 21576488
		Rule	NOT p53-A AND (NOT Sirt1 OR NOT Wip1) AND p53		p53_K can be activated in the absence of p53_A and the absence of Sirt1 or the absence of Wip1 and the presence of p53.
microRNA-21	miR-21	27	E2F1	E2F1 induces miR-21 expression	PMID: 34307154
		28	AKT	AKT induces miR-21 expression	PMID: 20814244
		29	PTENP1	PTENP1 inhibits miR-21 expression	PMID: 31656200
		Rule	(E2F1 AND AKT) OR NOT PTENP1		miR-21 can be activated in the presence of E2F1 and in the presence of AKT or in the absence of PTENP1.
Phosphatase and tensin homolog	PTEN	30	ATM	ATM induces PTEN expresion.	PMID: 25701194
		31	p53_K	p53_K induces PTEN expression.	PMID: 11545734
		32	miR-21	miR-21 inhibits PTEN.	PMID: 28374893
		33	SNAIL	SNAIL inhibits PTEN.	PMID: 18172008
		34	YY1	YY1 is a negative regulator of PTEN expression.	PMID: 32334006
		Rule	(ATM OR p53_K) AND NOT miR_21 AND NOT SNAIL AND NOT YY1		PTEN can be activated in the presence of ATM or in the presence of p53 and in the absence of miR-21 and in the absence of SNAIL and in

					the absence of YY1.
Pseudogene-derived long non-coding RNA PTENP1	PTEN P1	35	PTEN	PTEN induces PTENP1 expression.	PMID: 37894321
		Rule	PTEN		PTENP1 can be activated in the presece of PTEN.
AKT serine/threonine kinase 1	AKT	36	PTEN	PTEN inhibits AKT expression.	PMID: 34248141
		37	mTOR2	mTOR2 induces AKT expression.	PMID: 33850054
		Rule	NOT PTEN OR mTOR2		AKT can be activated when PTEN is absent or mTOR2 is present.
Polycomb complex protein BMI-1	BMI1	38	PTEN	PTEN inhibits BMI1 expression.	PMID: 19903340
		Rule	NOT PTEN		BMI1 can be activated when PTEN is not present.
Cyclin-dependent kinase inhibitor 1A	p21	39	p53-A	p53-A trigger activation of p21.	PMID: 31416295
		40	Myc	Myc is a negative regulator of p21 expression.	PMID: 27105536
		41	AKT	AKT is a negative regulator of p21 expression.	PMID: 11463845
		42	Caspase 3	Caspase 3 inhibits p21 expression.	PMID: 10022118
		43	BMI1	BMI1 is a negative regulator of p21 expression.	PMID: 24552182
		44	E2F1	E2F1 is a negative regulator of p21 expression.	PMID: 11076674
		45	AMPK	AMPK can induces p21 expression.	PMID: 27321921
		Rule	p53_A OR (NOT (Myc OR (AKT AND Caspase3) OR BMI1) AND NOT E2F1 AND AMPK)		p21 can be activated in the presence of p53-A or in the absence of Myc, or in the absence of AKT and in the absence of Caspase3 or in the absence of

					BMI1 and in the absence of E2F1 and in the presence of AMPK.
Raf kinase inhibitor protein	RKIP	46	p53_A	p53_A induces RKIP expression.	PMID: 23814485
		47	SNAIL	SNAIL inhibits RKIP expression.	PMID: 19538137
		Rule	p53_A AND NOT SNAIL		RKIP can be activated when p53-A is present and SNAIL is absent.
WEE1 G2 checkpoint kinase	Wee1	48	AKT	AKT is a negative regulator of Wee1 expression.	PMID: 35439317
		Rule	NOT AKT		Wee1 can be activated in the absence of AKT.
Sirtuin 1	Sirt1	49	E2F1	E2F1 is a positive regulator of Sirt1 expression.	PMID: 16892051
		Rule	E2F1		Sirt1 can be activated in the presence of E2F1.
Mg2+/Mn2+ Dependent 1D	Wip1	50	p53-A	Wip1 induces by p53-A.	PMID: 22405851
		Rule	p53-A		p53-A induces Wip1 expression.
RB transcriptional corepressor 1	RB	51	Cdk1-CycB	Cdc2-CycB inhibits RB expression.	PMID: 16618755
		52	Cdc25	Cdc25 is a negative regulator of RB expression.	PMID: 16618755
		53	Cdk2-CycA2	Cdk1-CycB inhibits RB expression.	PMID: 16618755
		Rule	NOT (Cdk1-CycB AND Cdc25 AND Cdk2-CycA2)		RB can be activated when Cdk1-CycB is absent and Cdc25 is absent and Cdk2-CycA2 is absent.
MYC proto-oncogene	Myc	54	E2F1	E2F1 trigger activation of Myc.	PMID: 18345030
		55	AMPK/MAPK	AMPK can induces Myc expression.	PMID: 29440228

		56	AKT	AKT is a positive regulator of Myc expression.	PMID: 27144349
		57	RB	RB is a negative regulator of Myc activity.	PMID: 27105536
		58	p21	p21 is a negative regulator of Myc activity.	PMID: 16923815
		Rule	(E2F1 OR AMPK OR AKT) AND NOT RB AND NOT p21		Myc can be activated in the presence of E2F1 or the presence of AMPK or in the presence of AKT and in the absence of RB and the absence of p21.
Mammalian target of rapamycin complex 1	mTOR 1	59	AKT	AKT induces mTOR1 expression.	PMID: 31746509
		60	ULK1	ULK1 directly inhibits mTORC1.	PMID: 31746509
		61	AMPK	AMPK directly inhibits mTOR1 expression.	PMID: 31746509
		Rule	AKT AND NOT (ULK1 AND AMPK)		mTOR1 can be activated when AKT is present and ULK1 is absent and AMPK is absent.
Mammalian target of rapamycin complex 2	mTOR 2	62	AKT	AKT induces mTOR2 expression.	PMID: 31746509
		63	PTEN	PTEN inhibits mTORC2 expression.	PMID: 36526374
		64	mTOR1	mTOR1 inhibits mTORC2 expression.	PMID: 31746509
		65	Sirt1	Sirt1 inhibits mTORC expression.	PMID: 20169165
		66	AMPK	AMPK directly inhibits mTOR2 expression.	PMID: 31746509
		Rule	AKT OR (NOT PTEN AND (NOT mTOR1 OR NOT Sirt1 OR NOT AMPK))		mTOR2 can be activated in the presence of AKT or in the absence of PTEN and in the absence of mTOR1 or in the absence of Sirt1 or in the absence of AMPK.
	E2F1	67	RB	RB is a negative regulator of E2F1 activity.	PMID: 23967231

E2F transcripition factor 1		68	Cdc25	Cdc25 is a positive regulator of E2F1 activity.	PMID: 22263797
		69	ATM	ATM is a positive regulator of E2F1 activity.	PMID: 11459832
		70	Sirt1	Sirt1 inhibits E2F1 expression.	PMID: 24020005
		71	PTEN	PTEN inhibits E2F1 expression.	PMID: 29108454
		72	Myc	Myc is a positive regulator of E2F1 expression.	PMID: 17784791
		Rule	(NOT RB AND ((Cdc25 AND ATM) OR NOT Sirt1 OR NOT PTEN)) OR Myc		E2F1 can be activated in the absence of RB and the presence of Cdc25A and the presence of ATM or the absence of Sirt1 or the absence of PTEN or the presence of Myc.
Cell division cycle 25c	Cdc25	73	ATM	ATM is a negative regulator of Cdc25 expression.	PMID: 11298456
		74	AMPK	AMPK is a negative regulator of Cdc25 expression.	PMID: 29467227
		75	Wee1	Wee1 inhibits Cdc25 expression.	PMID: 23751495
		76	PTEN	PTEN inhibits Cdc25 expression.	PMID: 29617654
		Rule	(NOT ATM OR NOT AMPK) AND NOT Wee1 AND NOT PTEN		Cdc25 can be activated in the absence of ATM or in the absence of AMPK and in the absence of Wee1 and in the absence of PTEN.
Cyclin-dependent kinase 2/Cyclin-A2	Cdk2-CycA2	77	Cdc25	Cdc25 is a positive regulator of Cdk2-CycA2	PMID: 7626805
		78	Wee1	Wee1 inhibits Cdk2-CycA2 expression.	PMID: 35045293
		79	p21	p21 inhibits Cdk2-CycA2 expression.	PMID: 7626805
		80	PTENP1	PTENP1 inhibits Cdk2-CycA2 expression.	PMID: 35655204
		Rule			

			Cdc25 AND NOT p21 AND NOT Wee1 AND NOT PTENP1		Cdk2-CycA2 can be activated when Cdc25 is presence and p21 is absent and Wee1 is absent and PTENP1 is absent.
Cyclin-dependent kinase 1/G2/mitotic-specific cyclin-B1	Cdk1-CycB	81	Cdc25	Cdc25 is a positive regulator of Cdc2-CycB activity.	PMID: 7626805
		82	Wee1	Wee1 inhibits Cdc2-CycB expression.	PMID: 35045293
		83	PTEN	PTEN inhibits Cdc2-CycB expression.	PMID: 37311884
		84	p21	p21 is a negative regulator of Cdc2-CycB activity.	PMID: 7626805
		Rule	Cdc25 AND NOT Wee1 AND NOT PTEN AND NOT p21		Cdk1-cycB can be activaed when Cdc25 is presence and Wee1 is absent and PTEN is absent and p21 is absent.
BCL2 apoptosis regulator	BCL2	85	E2F1	E2F1 ia a positive regulator of BCL2 activity.	PMID: 20411301
		86	p53_K	p53_K inhibits BCL2 expression.	PMID: 9419967
		87	PTEN	PTEN is a negative regulator of BCL2 activity.	PMID: 25111376
		Rule	(E2F1 AND NOT p53_K) OR NOT PTEN		BCL2 can be activated in the presence of E2F1 and in the absence of p53_k and in the absence of PTEN.
BCL2 associated X, apoptosis regulator	BAX	88	BCL2	BCL2 inhibits BAX expression.	PMID: 10713725
		89	p53_K	p53_K induces BAX expression.	PMID: 14963330
		Rule	NOT BCL2 AND p53-K		BAX can be activated in the absence of BCL2 and in the presence of p53-K.
Caspase-3	Caspas e 3	90	BCL2	BCL2 is a negative regulator of Caspase3.	PMID: 10409669

		91	p21	p21 is a negative regulator of Caspase3.	PMID: 9668108
		92	BAX	BAX is a positive regulator of Caspase3.	PMID: 10479688
		Rule	NOT (BCL2 AND p21) AND BAX		Caspase 3 can be activated in absence of Bcl2 and in the absence of p21 and in the presence of BAX.
Unc-51 like autophagy activating kinase 1	ULK1	93	mTOR2	mTOR2 inhibits ULK1 expression.	PMID: 23524951
		94	mTOR1	mTOR1 inhibits ULK1 expression.	PMID: 23524951
		95	AMPK	AMPK activates ULK1expression	PMID: 21258367
		Rule	NOT mTOR2 AND NOT mTOR1 AND AMPK		ULK1 can be activated in the absence of mTOR2 and in the absence of mTOR1 and in the presence of AMPK.
Nuclear factor NF-kappa-B p105 subunit	NFkB	96	PTEN	PTEN is a negative regulator of NFkB.	PMID: 11799112
		97	RKIP	RKIP inhibits NFkB.	PMID: 20043910
		Rule	NOT PTEN AND NOT RKIP		NFkB can be activated in the absence of PTEN and in the absence of RKIP.
YY1-associated factor 2	YY1	98	PTEN	PTEN is a negative regulator of YY1 expression.	PMID: 19483472
		99	NFkB	NFkB induces YY1 expression.	PMID: 17438126
		Rule	NOT PTEN AND NFkB		YY1 can be activated in the absence of PTEN and in the presence of NFkB.
Histone-lysine N-methyltransferase EZH2	EZH2	100	YY1	YY1 is a positive regulator of EZH2 activity.	PMID: 34065631
		Rule	YY1		EZH2 can be activated in the presence of EZH2.

Zinc finger protein SNAI1	SNAIL	101	AKT	AKT induces SNAIL expression.	PMID: 28934275
		102	EZH2	EZH2 induces SNAIL expression.	PMID: 28754964
		103	NFkB	NFkB induces SNAIL expression.	PMID: 17563753
		Rule	AKT AND EZH2 AND NFkB		SNAIL can be activated in the presence of AKT and in the presence of EZH2 and in the presence of NFkB.
Zinc finger E-box-binding homeobox 1	ZEB1	104	SNAIL	SNAIL induces ZEB1 expression.	PMID: 12161443
		Rule	SNAIL		ZEB1 can be activated in the presence of SNAIL.
Vimentin	VIM	105	SNAIL	SNAIL induces VIM expression.	PMID: 29623961
		106	ZEB1	ZEB1 induces VIM expression	PMID: 29623961
		Rule	SNAIL AND ZEB1		VIM can be activated in the presence of SNAIL and in the presence of ZEB1.
E-cadherin 1	CDH1	107	ZEB1	ZEB1 inhibits CDH1 expression.	PMID: 29623961
		Rule	NOT ZEB1		CDH1 can be activated in the absence of ZEB1.
DRUG RESISTANCE	DRUG RESISTANCE	108	E2F1	E2F1 can induces DRUG RESISTANCE.	PMID: 22871739
		109	BCL2	BCL2 can induces DRUG RESISTANCE.	PMID: 10432288
		Rule	E2F1 OR BCL2		DRUG RESISTANCE can be activated in the presence of E2F1 or in the presence of BCL2.
CELL CYCLE ARREST	CYCLE ARREST	110	p21	p21 is a positive regulator of CYCLE ARREST.	PMID: 27156098
		Rule	p21		Cycle arrest can be activated when p21 is presence.

SENESCENCE	SENESCENCE	111	p21	p21 is a positive regulator of SENESCENCE.	PMID: 27812865
		112	mTOR2	mTOR2 is a positive regulator of SENESCENCE.	PMID: 29190625
		Rule	p21 AND mTOR2		Senescence can be activated when p21 is presence and mTOR2 is presence.
AUTOPHAGY	AUTOPHAGY	113	ULK1	ULK1 is a positive regulator of AUTOPHAGY.	PMID: 35252196
		Rule	ULK1		Autophagy can be activated in the presence of ULK1.
APOPTOSIS	APOPTOSIS	114	Caspase 3	Caspase 3 is a positive regulator of APOPTOSIS.	PMID: 34940803
		115	ULK1	ULK1 is a Negative regulator of APOPTOSIS.	PMID: 30166400
		Rule	Caspase 3 AND NOT ULK1		Apoptosis can be activated in the presence of Caspase3 and in the absence of ULK1.
Epithelial-to-Mesenchymal Transition	EMT	116	CDH1	CDH1 is a Negative regulator of EMT.	PMID: 34727382
		117	VIM	VIM is a positive regulator of EMT.	PMID: 34638469
		Rule	NOT CDH1 AND VIM		EMT can be activated in the absence of CDH1 and in the presence of VIM.
