# Supplementary material for: LncRNA PTENP1/miR-21/PTEN Axis Modulates EMT and Drug Resistance in Cancer: Dynamic Boolean Modeling for Cell Fates in DNA Damage Response
Source: Int J Mol Sci. 2024 Jul 29;25(15):8264. doi: 10.3390/ijms25158264 (PMC11311614; doi:10.3390/ijms25158264)
Supplement: Supplementary file 1 [file ijms-25-08264-s001.zip › ijms-3117243-supplementary/Table S2.pdf]

Table S2

Table S2. Boolean network functional circuits and experimental observations. Cases not studied experimentally are indicated as Predicted.

**Positive feedback loos**	**References**
Myc/E2F1	[1]
Caspase3/p21	[2]
p53-A/p53-K	[3]
E2F1/ATM	[4]
RKIP/NFkB/SNAIL	[5]
Myc/p21	[6]
AKT/mTORC2	[7]
Cdc25/ATM	[8]
ULK1/mTOR1	[9]
PTEN/AKT/miR-21	[10]
PTEN/PTENP1/miR-21	Predicted
PTEN/E2F1/miR-21	Predicted
PTEN/BMI1/ATM	Predicted
PTEN/Cdc25/ATM	Predicted
PTEN/AKT/SNAIL	Predicted
PTEN/PTENP1/YY1	Predicted
PTEN/NFkB/YY1	Predicted
PTEN/NFkB/SNAIL	Predicted
**Negative feedback loop**	**References**
p53/MDM2	[11]
p53INP1/p53_A	[3]
E2F1/Sirt1	[12]
PTEN/E2F1/ATM	Predicted

References:

[1] H.A. Coller, J.J. Forman, A. Legesse-Miller, “Myc’ed messages”: myc induces transcription of E2F1 while inhibiting its translation via a microRNA polycistron, PLoS Genet 3 (2007) e146. https://doi.org/10.1371/journal.pgen.0030146.

[2] Y. Zhang, N. Fujita, T. Tsuruo, Caspase-mediated cleavage of p21Waf1/Cip1 converts cancer cells from growth arrest to undergoing apoptosis, Oncogene 18 (1999) 1131–1138. https://doi.org/10.1038/sj.onc.1202426.

[3] X.-P. Zhang, F. Liu, W. Wang, Two-phase dynamics of p53 in the DNA damage response, Proc Natl Acad Sci U S A 108 (2011) 8990–8995. https://doi.org/10.1073/pnas.1100600108.
[4] W.C. Lin, F.T. Lin, J.R. Nevins, Selective induction of E2F1 in response to DNA damage, mediated by ATM-dependent phosphorylation, Genes Dev 15 (2001) 1833–1844.
[5] K. Lin, S. Baritaki, L. Militello, G. Malaponte, Y. Bevelacqua, B. Bonavida, The Role of B-RAF Mutations in Melanoma and the Induction of EMT via Dysregulation of the NF-κB/Snail/RKIP/PTEN Circuit, Genes & Cancer 1 (2010) 409–420. https://doi.org/10.1177/1947601910373795.
[6] R.U. Jänicke, D. Sohn, F. Essmann, K. Schulze-Osthoff, The multiple battles fought by anti-apoptotic p21, Cell Cycle 6 (2007) 407–413. https://doi.org/10.4161/cc.6.4.3855.
[7] G. Yang, D.S. Murashige, S.J. Humphrey, D.E. James, A positive feedback loop between Akt and mTORC2 via SIN1 phosphorylation, Cell Reports 12 (2015) 937–943.
[8] C. Agarwal, A. Tyagi, R. Agarwal, Gallic acid causes inactivating phosphorylation of cdc25A/cdc25C-cdc2 via ATM-Chk2 activation, leading to cell cycle arrest, and induces apoptosis in human prostate carcinoma DU145 cells, Mol Cancer Ther 5 (2006) 3294–3302. https://doi.org/10.1158/1535-7163.MCT-06-0483.
[9] M. Holczer, B. Hajdú, T. Lőrincz, A. Szarka, G. Bánhegyi, O. Kapuy, A Double Negative Feedback Loop between mTORC1 and AMPK Kinases Guarantees Precise Autophagy Induction upon Cellular Stress, International Journal of Molecular Sciences 20 (2019) 5543. https://doi.org/10.3390/ijms20225543.
[10] D. Sayed, M. Abdellatif, AKT-ing via microRNA, Cell Cycle 9 (2010) 3233–3237. https://doi.org/10.4161/cc.9.16.12634.
[11] R. Lev Bar-Or, R. Maya, L.A. Segel, U. Alon, A.J. Levine, M. Oren, Generation of oscillations by the p53-Mdm2 feedback loop: a theoretical and experimental study, Proceedings of the National Academy of Sciences 97 (2000) 11250–11255.
[12] C. Wang, L. Chen, X. Hou, Z. Li, N. Kabra, Y. Ma, S. Nemoto, T. Finkel, W. Gu, W.D. Cress, J. Chen, Interactions between E2F1 and SirT1 regulate apoptotic response to DNA damage, Nat Cell Biol 8 (2006) 1025–1031. https://doi.org/10.1038/ncb1468.
