# Supplementary material for: LncRNA PTENP1/miR-21/PTEN Axis Modulates EMT and Drug Resistance in Cancer: Dynamic Boolean Modeling for Cell Fates in DNA Damage Response
Source: Int J Mol Sci. 2024 Jul 29;25(15):8264. doi: 10.3390/ijms25158264 (PMC11311614; doi:10.3390/ijms25158264)
Supplement: Supplementary file 1 [file ijms-25-08264-s001.zip › ijms-3117243-supplementary/Table S3.pdf]

Table S3

Table S3. The literature is rich with evidence detailing biochemical interactions that characterize these biological positive and negative circuits.

**Positive Circuit**	**Circuit Elements**	**Target**	**Direct/Indirect Interaction**	**References**
PTEN/PTENP1/miR-21	PTEN	PTENP1	Direct activation	[1]
	PTENP1	miR-21	Direct inhibition	[2]
	miR-21	PTEN	Direct inhibition	[2]
PTEN/E2F1/miR-21	PTEN	E2F1	Direct inhibition	[3]
	E2F1	miR-21	Direct activation	[4]
	miR-21	PTEN	Direct inhibition	[2]
PTEN/BMI1/ATM	PTEN	BMI1	Direct inhibition	[5]
	BMI1	ATM	Direct inhibition	[6]
	ATM	PTEN	Direct activation	[7]
PTEN/Cdc25/ATM	PTEN	Cdc25	Direct inhibition	[8]
	Cdc25	ATM	Direct inhibition	[9]
	ATM	PTEN	Direct activation	[7]
PTEN/AKT/SNAIL	PTEN	AKT	Direct inhibition	[10]
	AKT	SNAIL	Direct activation	[11]
	SNAIL	PTEN	Direct inhibition	[12]
PTEN/PTENP1/YY1	PTEN	PTENP1	Direct activation	[2]
	PTENP1	YY1	Direct inhibition	[13]
	YY1	PTEN	Direct inhibition	[14]
PTEN/NFkB/YY1	PTEN	NFkB	Direct inhibition	[15]
	NFkB	YY1	Direct activation	[16]
	YY1	PTEN	Direct inhibition	[14]
PTEN/NFkB/SNAIL	PTEN	NFkB	Direct inhibition	[15]
	NFkB	SNAIL	Direct activation	[17]
	SNAIL	PTEN	Direct inhibition	[12]
**Negative Circuit**	**Circuit Elements**	**Target**	**Direct/Indirect Interaction**	**References**
PTEN/E2F1/ATM	PTEN	E2F1	Direct inhibition	[3]
	E2F1	ATM	Direct activation	[18]
	ATM	PTEN	Direct activation	[18]

References:

[1] G. Yu, W. Yao, K. Gumireddy, A. Li, J. Wang, W. Xiao, K. Chen, H. Xiao, H. Li, K. Tang, Z. Ye, Q. Huang, H. Xu, Pseudogene PTENP1 Functions as a Competing Endogenous RNA to Suppress Clear-Cell Renal Cell Carcinoma Progression, Molecular Cancer Therapeutics 13 (2014) 3086–3097. https://doi.org/10.1158/1535-7163.MCT-14-0245.

[2] L. Gao, W. Ren, L. Zhang, S. Li, X. Kong, H. Zhang, J. Dong, G. Cai, C. Jin, D. Zheng, K. Zhi, PTENp1, a natural sponge of miR-21, mediates PTEN expression to inhibit the proliferation of oral squamous cell carcinoma, Mol Carcinog 56 (2017) 1322–1334. https://doi.org/10.1002/mc.22594.

[3] P. Malaney, E. Palumbo, J. Semidey-Hurtado, J. Hardee, K. Stanford, J.J. Kathiriya, D. Patel, Z. Tian, D. Allen-Gipson, V. Davé, PTEN Physically Interacts with and Regulates E2F1-mediated Transcription in Lung Cancer, Cell Cycle 17 (2018) 947–962. https://doi.org/10.1080/15384101.2017.1388970.

[4] S. Qin, J. Xu, Y. Yi, S. Jiang, P. Jin, X. Xia, F. Ma, Transcription Factors and Methylation Drive Prognostic miRNA Dysregulation in Hepatocellular Carcinoma, Front. Oncol. 11 (2021). https://doi.org/10.3389/fonc.2021.691115.

[5] C. Fan, L. He, A. Kapoor, A.P. Rybak, J. De Melo, J.-C. Cutz, D. Tang, PTEN inhibits BMI1 function independently of its phosphatase activity, Mol Cancer 8 (2009) 98. https://doi.org/10.1186/1476-4598-8-98.

[6] V. Ginjala, K. Nacerddine, A. Kulkarni, J. Oza, S.J. Hill, M. Yao, E. Citterio, M. van Lohuizen, S. Ganesan, BMI1 is recruited to DNA breaks and contributes to DNA damage-induced H2A ubiquitination and repair, Molecular and Cellular Biology 31 (2011) 1972–1982.

[7] J.-H. Chen, P. Zhang, W.-D. Chen, D.-D. Li, X.-Q. Wu, R. Deng, L. Jiao, X. Li, J. Ji, G.-K. Feng, Y.-X. Zeng, J.-W. Jiang, X.-F. Zhu, ATM-mediated PTEN phosphorylation promotes PTEN nuclear translocation and autophagy in response to DNA-damaging agents in cancer cells, Autophagy 11 (2015) 239–252. https://doi.org/10.1080/15548627.2015.1009767.

[8] R. Zhang, L. Zhu, L. Zhang, A. Xu, Z. Li, Y. Xu, P. He, M. Wu, F. Wei, C. Wang, PTEN enhances G2/M arrest in etoposide-treated MCF-7 cells through activation of the ATM pathway, Oncol Rep 35 (2016) 2707–2714. https://doi.org/10.3892/or.2016.4674.

[9] D. Verduzco, J.S. Dovey, A.A. Shukla, E. Kodym, B.A. Skaug, J.F. Amatruda, Multiple isoforms of CDC25 oppose ATM activity to maintain cell proliferation during vertebrate development, Mol Cancer Res 10 (2012) 1451–1461. https://doi.org/10.1158/1541-7786.MCR-12-0072.

[10] C. Blanco-Aparicio, O. Renner, J.F.M. Leal, A. Carnero, PTEN, more than the AKT pathway, Carcinogenesis 28 (2007) 1379–1386. https://doi.org/10.1093/carcin/bgm052.

[11] J. Dong, B. Zhai, W. Sun, F. Hu, H. Cheng, J. Xu, Activation of phosphatidylinositol 3-kinase/AKT/snail signaling pathway contributes to epithelial-mesenchymal transition-induced multi-drug resistance to sorafenib in hepatocellular carcinoma cells, PLoS One 12 (2017) e0185088. https://doi.org/10.1371/journal.pone.0185088.

[12] M. Escrivà, S. Peiró, N. Herranz, P. Villagrasa, N. Dave, B. Montserrat-Sentís, S.A. Murray, C. Francí, T. Gridley, I. Virtanen, A. García de Herreros, Repression of PTEN Phosphatase by Snail1 Transcriptional Factor during Gamma Radiation-Induced Apoptosis, Mol Cell Biol 28 (2008) 1528–1540. https://doi.org/10.1128/MCB.02061-07.

[13] C. Yi, G. Li, W. Wang, Y. Sun, Y. Zhang, C. Zhong, D.B. Stovall, D. Li, J. Shi, G. Sui, Disruption of YY1-EZH2 Interaction Using Synthetic Peptides Inhibits Breast Cancer Development, Cancers (Basel) 13 (2021) 2402. https://doi.org/10.3390/cancers13102402.

[14] B. Bonavida, Chapter 13 - The role of YY1 in drug resistant cancer: Involvement of the YY1/PTEN/PP2A/H2Ax/Rad51 axis, in: B. Bonavida (Ed.), YY1 in the Control of the Pathogenesis and Drug Resistance of Cancer, Academic Press, 2021: pp. 225–242. https://doi.org/10.1016/B978-0-12-821909-6.00006-7.

[15] L. Gu, N. Zhu, H.W. Findley, M. Zhou, Loss of PTEN Expression Induces NF-kB Via PI3K/Akt Pathway Involving Resistance to Chemotherapy in Acute Lymphoblastic Leukemia Cell Lines., Blood 104 (2004) 4438. https://doi.org/10.1182/blood.V104.11.4438.4438.

[16] H. Wang, E. Hertlein, N. Bakkar, H. Sun, S. Acharyya, J. Wang, M. Carathers, R. Davuluri, D.C. Guttridge, NF-κB Regulation of YY1 Inhibits Skeletal Myogenesis through Transcriptional Silencing of Myofibrillar Genes, Mol Cell Biol 27 (2007) 4374–4387. https://doi.org/10.1128/MCB.02020-06.

[17] B. Bonavida, Linking Autophagy and the Dysregulated NFκB/SNAIL/YY1/RKIP/PTEN Loop in Cancer: Therapeutic Implications, Crit Rev Oncog 23 (2018) 307–320. https://doi.org/10.1615/CritRevOncog.2018027212.

[18] W.C. Lin, F.T. Lin, J.R. Nevins, Selective induction of E2F1 in response to DNA damage, mediated by ATM-dependent phosphorylation, Genes Dev 15 (2001) 1833–1844.
