# Supplementary material for: LncRNA PTENP1/miR-21/PTEN Axis Modulates EMT and Drug Resistance in Cancer: Dynamic Boolean Modeling for Cell Fates in DNA Damage Response
Source: Int J Mol Sci. 2024 Jul 29;25(15):8264. doi: 10.3390/ijms25158264 (PMC11311614; doi:10.3390/ijms25158264)
Supplement: Supplementary file 1 [file ijms-25-08264-s001.zip › ijms-3117243-supplementary/Table S4.pdf]

Table S4

Below are comprehensive descriptions of each circuit, including their perturbations and outcomes. Perturbations leading to EMT concurrent with cell cycle arrest, senescence, autophagy, or apoptosis are highlighted in bold fonts.

Table S4. Analysis of Gain-of-Function (GoF) and Loss-of-Function (LoF) Perturbations in Newly Identified Positive and Negative Circuits.

Positive Circuit	Perturbations	Phenotypes
PTEN/PTENp1/miR-21	KO/KO/KO	EMT
	E1/KO/KO	Cell cycle arrest/Senescence/Autophagy/Apoptosis
	KO/E1/KO	Drug Resistance
	KO/KO/E1	EMT
	E1/E1/KO	Cell cycle arrest/Senescence/Autophagy/Apoptosis
	**E1/KO/E1**	**Cell cycle arrest/Senescence/Autophagy/Apoptosis and EMT**
	KO/E1/E1	Drug Resistance
	E1/E1/E1	Cell cycle arrest/Senescence/Autophagy/Apoptosis
**Positive Circuit**	**Perturbations**	**Phenotypes**
	KO/KO/KO	Drug Resistance/EMT
	E1/KO/KO	Cell cycle arrest/Senescence/Autophagy/Apoptosis
	KO/E1/KO	Drug Resistance/EMT

PTEN/E2F1/miR-21	KO/KO/E1	Drug Resistance/EMT
	E1/E1/KO	Autophagy/Apoptosis
	E1/KO/E1	Cell cycle arrest/Senescence/Autophagy/Apoptosis
	KO/E1/E1	Drug Resistance/EMT
	E1/E1/E1	Autophagy/Apoptosis
**Positive Circuit**	**Perturbations**	**Phenotypes**
PTEN/BMI1/ATM	KO/KO/KO	EMT
	KO/E1/KO	EMT
	**KO/KO/E1**	**Senescence/ Drug Resistance/EMT**
	E1/KO/E1	Cell cycle arrest/Senescence/Autophagy/Apoptosis
	**KO/E1/E1**	**Senescence/ Drug Resistance/EMT**
	E1/E1/E1	Cell cycle arrest/Senescence/Autophagy/Apoptosis
**Positive Circuit**	**Perturbations**	**Phenotypes**
PTEN/Cdc25/ATM	KO/E1/KO	EMT
	**KO/KO/E1**	**Senescence/ Drug Resistance/EMT**
	E1/KO/E1	Cell cycle arrest/Senescence/Autophagy/Apoptosis
	**KO/E1/E1**	**Senescence/ Drug Resistance/EMT**
	E1/E1/E1	Cell cycle arrest/Senescence/Autophagy/Apoptosis
**Positive Circuit**	**Perturbations**	**Phenotypes**
PTEN/AKT/SNAIL	KO/KO/KO	Drug Resistance

	E1/KO/KO	Cell cycle Arrest/Autophagy
	KO/E1/KO	Drug Resistance
	KO/KO/E1	EMT
	E1/E1/KO	Senescence/Apoptosis
	**E1/KO/E1**	**Cell cycle Arrest /Autophagy and EMT**
	KO/E1/E1	EMT
	**E1/E1/E1**	**Senescence /Apoptosis and EMT**
**Positive Circuit**	**Perturbations**	**Phenotypes**
PTEN/PTENp1/YY1	KO/KO/KO	Drug Resistance
	E1/KO/KO	Cell cycle arrest/Senescence/Autophagy/Apoptosis
	KO/E1/KO	Drug Resistance
	KO/KO/E1	EMT
	E1/E1/KO	Cell cycle arrest/Senescence/Autophagy/Apoptosis
	**E1/KO/E1**	**Cell cycle arrest/Senescence/Autophagy/Apoptosis and EMT**
	KO/E1/E1	EMT
	**E1/E1/E1**	**Cell cycle arrest/Senescence/Autophagy/Apoptosis and EMT**
**Positive Circuit**	**Perturbations**	**Phenotypes**
PTEN/NFkB/YY1	KO/KO/KO	Drug Resistance
	E1/KO/KO	Cell cycle arrest/Senescence./Autophagy/Apoptosis

	KO/E1/KO	Drug Resistance
	KO/KO/E1	Drug Resistance
	E1/E1/KO	Cell cycle arrest/Senescence/Autophagy/Apoptosis
	E1/KO/E1	Cell cycle arrest/Senescence/Autophagy/Apoptosis
	KO/E1/E1	Drug Resistance/EMT
	**E1/E1/E1**	**Cell cycle arrest/Senescence/Autophagy/Apoptosis and EMT**
**Positive Circuit**	**Perturbations**	**Phenotypes**
PTEN/NFkB/SNAIL	KO/KO/KO	Drug Resistance
	E1/KO/KO	Cell cycle arrest/Senescence/Autophagy/Apoptosis
	KO/E1/KO	Drug Resistance
	KO/KO/E1	Drug Resistance/EMT
	E1/E1/KO	Cell cycle arrest/Senescence/Autophagy/Apoptosis
	**E1/KO/E1**	**Cell cycle arrest/Senescence/Autophagy/Apoptosis and EMT**
	KO/E1/E1	Drug Resistance/EMT
	**E1/E1/E1**	**Cell cycle arrest/Senescence/Autophagy/Apoptosis and EMT**
**Negative Circuit**	**Perturbations**	**Phenotypes**

PTEN/E2F1/ATM	KO/E1/KO	Drug Resistance/EMT
	KO/KO/E1	Drug Resistance/EMT
	E1/E1/KO	Drug Resistance
	E1/KO/E1	Cell cycle arrest/Senescence/Autophagy/Apoptosis
	KO/E1/E1	Drug Resistance/EMT
	E1/E1/E1	Autophagy/Apoptosis

The findings are outlined in Table S4. Within the PTEN/PTENp1/miR-21 circuit, various perturbations elicit distinct cellular responses. Knocking out all three molecules (KO/KO/KO) induces a transition known as EMT. Conversely, when PTEN is overexpressed alone (E1/KO/KO), it triggers diverse cellular reactions, including cell cycle arrest, senescence, autophagy, and apoptosis. Depleting PTEN and miR-21 while overexpressing PTENP1 (KO/E1/KO) results in acquired resistance, while simultaneous knockout of PTEN and PTENP1 alongside miR-21 overexpression (KO/KO/E1) also leads to EMT. Notably, the combined overexpression of PTEN and PTENP1 with miR-21 knockout (E1/E1/KO) drives cell cycle arrest, senescence, autophagy, and apoptosis. These findings underscore the critical roles these molecules play in cellular fate decisions and highlight their potential as therapeutic targets.

In another positive circuit involving PTEN/E2F1/miR-21, the knockout of all three molecules (KO/KO/KO) leads to acquired resistance and triggers EMT. Conversely, overexpressing PTEN alone (E1/KO/KO) induces a spectrum of cellular outcomes, including cell cycle arrest, senescence, autophagy, and apoptosis. Depleting PTEN and miR-21, while overexpressing E2F1 (KO/E1/KO), results in resistance and induces EMT. Similarly, when miR-21 overexpression is combined with E2F1 and PTEN knockout (KO/KO/E1), it also induces resistance and EMT. Notably, the overexpression of PTEN alongside E2F1 (E1/E1/KO) leads to autophagy and apoptosis. Similarly, when PTEN and miR-21 are overexpressed in combination with E2F1 knockout (E1/KO/E1), it drives cell cycle arrest, senescence, autophagy, and apoptosis. These findings highlight the complex interplay within this circuit and

provide insights into the diverse cellular responses mediated by PTEN, E2F1, and miR-21. For more detail, refer to Table S4.

In the PTEN/BMI1/ATM circuit, distinct perturbations lead to diverse cellular outcomes. Knocking out all three molecules (KO/KO/KO) induces EMT. Conversely, when PTEN is overexpressed alone (E1/KO/KO), it results in oscillatory behavior. Depleting PTEN and ATM, while overexpressing BMI1 (KO/E1/KO), leads to EMT. Similarly, when ATM is overexpressed alongside PTEN and BMI1 knocking out (KO/KO/E1), it induces senescence and EMT. Notably, overexpression of PTEN and ATM, along with BMI1 knockout (E1/KO/E1), drives cell cycle arrest, senescence, autophagy, and apoptosis. Similarly, when BMI1 and ATM overexpression are combined with PTEN knockout (KO/E1/E1), it induces senescence and EMT. These results underscore the intricate dynamics within this circuit and provide insights into the diverse cellular responses mediated by PTEN, BMI1, and ATM.

The PTEN/Cdc25/ATM circuit is a complex interplay of molecules, each with a specific role in triggering diverse cellular outcomes. Knocking out all three molecules (KO/KO/KO) induces oscillatory behavior while overexpressing PTEN alone (E1/KO/KO) leads to oscillatory responses. Inducing EMT requires depleting PTEN and ATM while overexpressing Cdc25 (KO/E1/KO), and senescence and EMT are triggered when PTEN and Cdc25 are knocked out alongside ATM overexpression (KO/KO/E1). Overexpression of PTEN and Cdc25 along with ATM knockout (E1/E1/KO) drives oscillatory behavior, and overexpressing PTEN and ATM in combination with Cdc25 knockout (E1/KO/E1) results in cell cycle arrest, senescence, autophagy, and apoptosis. These findings shed light on the intricate workings of this circuit and provide crucial insights into the diverse cellular responses mediated by PTEN, Cdc25, and ATM. For more detail, see Table S4.

In the PTEN/PTENp1/YY1 circuit, knocking out all three molecules (KO/KO/KO) induces drug resistance. Conversely, when PTEN is overexpressed alone (E1/KO/KO), it triggers a range of cellular responses, including cell cycle arrest, senescence, autophagy, and apoptosis. Depleting PTEN and YY1, while overexpressing PTENP1 (KO/E1/KO), leads to resistance. Similarly, when PTEN and PTENP1 are knocked out alongside YY1 overexpression (KO/KO/E1), it induces EMT. Notably, overexpression of PTEN and PTENP1, along with YY1 knockout (E1/E1/KO), drives cell cycle arrest, senescence, autophagy, and apoptosis. Similarly,

when PTEN and YY1 are overexpressed in combination with PTENP1 knockout (E1/KO/E1), it leads to cell cycle arrest, senescence, autophagy, and apoptosis, potentially attenuating EMT.

In the PTEN/NFkB/YY1 circuit, various alterations yield diverse cellular outcomes. Knocking out all three molecules (KO/KO/KO) induces drug resistance. However, when PTEN is overexpressed alone (E1/KO/KO), it triggers a range of cellular responses, including cell cycle arrest, senescence, autophagy, and apoptosis. Depleting PTEN and YY1, while overexpressing NFkB (KO/E1/KO), also results in resistance. Similarly, when PTEN and NFkB are knocked out with YY1 overexpression (KO/KO/E1), it likewise leads to resistance. Conversely, overexpressing PTEN and NFkB with YY1 knockout (E1/E1/KO) induces cell cycle arrest, senescence, autophagy, and apoptosis. Likewise, overexpression of PTEN and YY1, along with NFkB knockout (E1/KO/E1), leads to cell cycle arrest, senescence, autophagy, and apoptosis, potentially reducing EMT. For more details, refer to Table S4.

he PTEN/NFkB/SNAIL circuit plays a significant role in drug resistance induction. When all three molecules are knocked out (KO/KO/KO), it results in drug resistance. In contrast, overexpressing PTEN alone (E1/KO/KO) triggers various cellular responses such as cell cycle arrest, senescence, autophagy, and apoptosis. Depleting PTEN and SNAIL while overexpressing NFkB (KO/E1/KO) also leads to resistance. Similarly, inducing EMT occurs when PTEN and NFkB are knocked out, and SNAIL is overexpressed (KO/KO/E1). However, overexpression of PTEN and NFkB with SNAIL knockout (E1/E1/KO) promotes cell cycle arrest, senescence, autophagy, and apoptosis. Conversely, PTEN and SNAIL overexpression, accompanied by NFkB knockout (E1/KO/E1), leads to cell cycle arrest, senescence, autophagy, apoptosis, and EMT. For further details, refer to Table S4.

The negative circuit involving PTEN, E2F1, and ATM confidently responds to different changes, yielding a variety of cellular responses. Knocking out all three molecules (KO/KO/KO) or overexpressing PTEN while knocking out E2F1 and ATM (E1/KO/KO) results in oscillatory patterns. When PTEN is knocked out alongside E2F1 overexpression (KO/E1/KO) or vice versa (KO/KO/E1), drug resistance and EMT occur. Overexpressing PTEN in combination with E2F1 overexpression (E1/E1/KO) leads to drug resistance while overexpressing PTEN in combination with

ATM overexpression (E1/KO/E1) induces cell cycle arrest, senescence, autophagy, and apoptosis. Conversely, when E2F1 and ATM are overexpressed along with PTEN knockout (KO/E1/E1), drug resistance and EMT are observed. Finally, overexpressing all three molecules (E1/E1/E1) induces autophagy and apoptosis, highlighting the complexity of this regulatory network.
