# Supplementary material for: LncRNA PTENP1/miR-21/PTEN Axis Modulates EMT and Drug Resistance in Cancer: Dynamic Boolean Modeling for Cell Fates in DNA Damage Response
Source: Int J Mol Sci. 2024 Jul 29;25(15):8264. doi: 10.3390/ijms25158264 (PMC11311614; doi:10.3390/ijms25158264)
Supplement: Supplementary file 1 [file ijms-25-08264-s001.zip › ijms-3117243-supplementary/Table S5.pdf]

Table S5

Table S5: Observed Oscillations in Perturbation Analysis of Three Circuits: Two Positive (PTEN/BMI1/ATM and PTEN/Cdc25/ATM) and One Negative (PTEN/E2F1/ATM).

**Positive Circuits**	**Perturbations**	**Phenotypes**
PTEN/BMI1/ATM	E1/KO/KO	Oscillations
	E1/E1/KO	Oscillations
PTEN/Cdc25/ATM	KO/KO/KO	Oscillations
	E1/KO/KO	Oscillations
	E1/E1/KO	Oscillations
**Negative Circuit**	**Perturbations**	**Phenotypes**
PTEN/E2F1/ATM	KO/KO/KO	Oscillations
	E1/KO/KO	Oscillations
